# Blockchain technology for supply chain traceability: A game-theoretic analysis between e-platforms

**DOI:** 10.1371/journal.pone.0297978

**Published:** 2024-04-25

**Authors:** Qian Chen, Xuejian Yang, Dan Yang, Sen Liu, Chuchu Liao

**Affiliations:** 1 School of Management, Hefei University of Technology, Hefei, China; 2 Dali Tobacco Company of Yunnan Province, Dali, China; 3 International College, Dhurakij Pundit University, Bangkok, Thailand; 4 School of Logistics and Management Engineering, Yunnan University of Finance and Economics, Kunming, China; 5 Yunnan Key Laboratory of Service Computing, Kunming, China; University of Victoria / Universiti Teknologi Malaysia /, CANADA

## Abstract

In view of the rapid development and application of blockchain technology, this paper considers a secondary supply chain system consisting of a single upstream supplier and a downstream e-tailer that implements blockchain technology and a traditional e-tailer that does not implement blockchain technology. We establish the demand function of two channels based on consumers’ sensitivity to the blockchain and use the Stackelberg game model to compare and analyse the e-tailers’ profits from the two channels. For the basic properties, interestingly, we find that there exists a critical threshold on the cost sensitivity to effort that helps each e-tailer decide whether to implement effort. If the cost sensitivity to effort is high, the two-sided e-tailers will reduce their effort as much as possible to obtain greater profits. Conversely, if the cost sensitivity to effort is low, they will increase their effort to obtain more benefits. We also discuss the role of blockchain technology in competition between e-tailers and analyse the impact of the product brand effect brought by the traceability characteristic of blockchain on the competition between e-tailers. To check the robustness of the core results and to investigate different forms of supply chain configurations, this paper further develops the analysis of the supplier entering agency agreements with two-sided e-tailers. Under this scenario, the supplier sells their products directly to consumers through two-sided e-platforms and shares revenue with e-tailers as platform agency fees. These core ideas remain valid in the extended model.

## 1. Introduction

Dual-channel competition has always been the focus of research in the field of supply chains, but the subjects of competition are not the same. In recent years, due to the development and application of blockchain technology [1], as well as consumers’ demands for product authenticity [2, 3], some retailers have been prompted to adopt blockchain technology to provide consumers with product source traceability services, including Walmart and JD. Although the application of this technology is mostly on a small scale, it still has a certain impact on traditional retail platforms [4]. As a result, a new competitive model is formed in the market: horizontal competition between a new retail platform that provides blockchain technology and a traditional retail platform that does not use blockchain technology.

In the retail industry, lack of supply chain traceability is a challenge to check that products are genuine and safe. For this pain point, blockchain technology has the characteristics of traceability; it helps to improve the safety and authenticity of products and solve the consumer’s trust crisis in the product [4–6]. For example, the product traceability service brought by blockchain technology has had a positive spillover effect on Walmart’s brand [7]. Although related research has found that the traceability of blockchain can indeed bring consumer stickiness, which we call the brand value effect (JD.com, 2020), the traceability system will also bring costs per unit of product, such as product information entry and information maintenance on the chain [8, 9]. Therefore, traditional retail platforms that do not use blockchain technology can still compete with blockchain retail platforms through other marketing efforts. In summary, motivated by the observed real-world industrial practice on e-platform operations for product sales services and the importance of product traceability in the blockchain era, this paper will study the horizontal competition between blockchain platforms and traditional platforms.

Although horizontal competition for e-commerce exists and there is an urgency to address the aforementioned issues, previous literature has focused on investigating the inner mechanism within a particular blockchain platform [10, 11]. The horizontal competition between e-tailers in a dual-channel supply chain based on blockchain technology remains largely unexplored. In this paper, we aim to fill this research gap by analysing the competition between electronic platforms from the perspective of the brand value effect brought by the traceability of the blockchain.

Specifically, we address the following research questions.

- What factors affect the profitability of dual-channel supply chain members?
- What factors affect the competition between the two e-platforms?
- To check the robustness of the core results, is it possible to obtain similar results in the extended model?

To answer these questions and fill the void in the literature, we develop a dual-channel platform competition game-theoretic duopoly economic model based on the heterogeneity of consumers’ sensitivity to blockchain technology, in which the two electronic platforms compete in the scenarios of using blockchain technology (the new e-tailer) and not using blockchain technology (the traditional e-tailer). Under both scenarios, competing e-tailers determine their platform effort based on the manufacturer’s wholesale prices and ultimately obtain the optimal solution for the profits of dual-channel supply chain members. We provide numerical simulation to support our theoretical findings, enhance the practical implications, and provide management insights into electronic platform competition in the context of blockchain.

To the best of our knowledge, this paper is the first to establish a demand function based on consumers’ sensitivity and the heterogeneity of blockchain technology. Meanwhile, this paper is also the first paper in the literature that analytically explores the product traceability between two competing e-platforms for product sales services in the application of blockchain technology. Specifically, our study shows the following insights. (i) For the basic properties, interestingly, we find that there exists a critical threshold on the cost sensitivity to effort that helps each e-tailer decide whether to implement effort. If the cost sensitivity to effort is high, two-sided e-tailers will reduce their effort as much as possible to obtain greater profits. Conversely, if the cost sensitivity to effort is low, they will increase their effort to obtain more benefits. (ii) In the comparison of optimal profit, the results show that when the blockchain-sensitive consumer market has a relatively small market share but the brand value effect with blockchain is large, if the cost sensitivity to the effort is in a small range, the new e-tailer implementing blockchain obtains more benefits. However, when the blockchain-sensitive consumer market accounts for a relatively large market, no matter how the brand value effect with blockchain and the cost sensitivity to the effort are valued within their range, the profit level of the new e-tailer is always greater than that of the traditional e-tailer.

In addition, to check the robustness of the conclusions, we investigate the extended model by considering that the supplier enters agency selling agreements with e-platforms. In this scenario, the supplier can sell products directly to consumers through the blockchain e-platform and the traditional e-platform and share revenue with two-sided e-tailers as platform agency fees. Based on the model and numerical analysis, we find that the above core ideas remain valid in the extended model. Finally, the most important finding is that e-tailers are always more profitable in the agency model regardless of the scope of the cost sensitivity to effort. In particular, when the cost sensitivity to effort is low enough, the agency model may be a more efficient way to improve the benefits of supply chain members.

The remainder of this paper is organized as follows: The next section reviews the literature from three streams. Section 3 describes the model. Section 4 analyses the properties of decision variables and compares the profits of e-tailers. Section 5 verifies robustness of core results. Section 6 provides numerical experiments. Section 7 discusses the findings and provides managerial insights, and Section 8 is the conclusion.

## 2. Literature review

Our research is closely related to three streams of literature: literature on (i) blockchain technology in the supply chain, (ii) competition in the dual-channel supply chain, and (iii) heterogeneous consumers. We will consider the theme of heterogeneous consumers in a dual-channel supply chain competition based on the context of blockchain technology, and provide an overview of the third aspect above, following the logic of moving from surface to line to point. Next, we describe how our research relates to the literature in these areas.

### 2.1 Blockchain technology in supply chain

At present, the exploration of “blockchain + supply chain” is mainly presented in the literature around three major characteristics of the blockchain: the reduction of the bullwhip effect, smart contracts, and traceability. For example, Cole et al. [12] comprehensively and objectively considered the impact of various characteristics of the blockchain on the operation and management of the supply chain. They posit that whether to adopt blockchain technology is based on the characteristics of the supply chain. From the perspective of operations management, relevant scholars have tried to layout a research agenda for the OM field; they consider the impact of blockchain technology on classic operations management issues (such as the bullwhip effect, its causes and mitigation strategies) and further explore new business models to solve this problem, which is a focus of scholars and companies. With further research, related scholars have begun to quantitatively explore the influence of blockchain technology on the bullwhip effect. On the one hand, Giovanni [13] used game theory methods to compare the profit of supply chain members under the use of blockchain and traditional situations and finds that blockchain technology can reduce business risks and transaction costs. On the other hand, Chang et al. [14] clarified how the adoption of blockchain technology affects the best ordering decisions and the corresponding best profits. The results show that the adoption of blockchain technology is not always profitable.

A smart contract is a computer protocol designed to facilitate, verify, or enforce the negotiation or execution of a contract. Specifically, the smart contract monitors the operations fulfilment as written in the blockchain, which allows us to confirm the planned process progress and to identify deviations. A smart contract typically stores rules and policies for negotiating terms and actions between parties. It automatically verifies that contractual terms have been met and executes transactions [15]. In view of this characteristic, Dolgui et al. [16] developed a model for blockchain-driven smart contract design and execution control and focus on the application of smart contracts in the field of “blockchain + supply chain”. Moreover, through quantitative research, Giovanni [13] did find that smart revenue-sharing contracts are very efficient digital mechanisms and guarantee better performance than the sole blockchain.

The Hyperledger Fabric blockchain-based data module ensures the authenticity and traceability of the data source [17, 18]. The application of traceability can create transparency in supply chains [19]. With the specific company case, Bumblauskas et al. [20] aimed to illustrate the application of blockchain traceability technology in egg production and supply chain distribution systems from farms to consumers. Some scholars in related fields use qualitative methods to demonstrate the value of blockchain traceability to the supply chain in different contexts [21–23]; others use quantitative methods to compare and discover that the supply chain value brought by blockchain traceability is higher than that of the traditional supply chain [24, 25]. However, most of the articles found that whether blockchain traceability can always be beneficial requires supply chain members to weigh the relevant factors. Specifically, Zhang et al. explore how the grey market influences manufacturers’ decisions to adopt blockchain by exploring whether and when manufacturers’ adoption of blockchain discourages or facilitates grey marketers [26]. Xu et al. Consider a manufacturer’s operational decisions in the presence of remanufacturing and blockchain [27]. Wu et al. focus on the optimal strategy for the adoption of blockchain technology in the supply chain of fresh produce, taking into account consumer preferences for traceability information [28]. Zhang et al. investigate the impact of different blockchain application scenarios on dual-channel supply chain decisions and further analyses the conditions for blockchain adoption [29]. Zhang et al. consider static versus dynamic pricing for blockchain technology platforms versus traditional platforms in the presence of network effects in a competitive environment [30]. However, different from the above research, Kouhizadeh et al. [31] discuss the application barriers of blockchain technology in the supply chain from the perspective of traceability. To show the difference more clearly, we summarize the previous literature regarding blockchain technology in supply chains. As shown in Table 1.

**Table 1:** Summary of the related literature regarding blockchain technology in supply chains.

Literature	Research methods	The characteristics of blockchain			Involves dual-channel competition
		Reduce the bullwhip effect	Smart contracts	Transparent and traceability	
Cole et al. [12]	Qualitative	√	√	√	
Giovanni [13]	Quantitative	√	√		
Chang et al. [14]	Quantitative	√			
Saberi et al. [15]	Qualitative		√		
Dolgui et al. [16]	Quantitative		√		
Song et al. [17]	Quantitative			√	
Qiao et al. [18]	Quantitative			√	
Sunny et al. [19]	Qualitative			√	
Bumblauskas et al. [20]	Qualitative			√	
Mukherjee et al. [23]	Qualitative			√	
Pournader et al. [22]	Qualitative			√	
Hastig and Sodhi [21]	Qualitative			√	
Liu et al. [24]	Quantitative	√		√	
Liu et al. [25]	Quantitative			√	
Zhang et al. [26]	Quantitative			√	
Xu et al. [27]	Quantitative			√	
Wu et al. [28]	Quantitative			√	
Zhang et al. [29]	Quantitative			√	
Zhang et al. [30]	Quantitative			√	
Kouhizadeh et al. [31]	Qualitative			√	
Our study	Quantitative			√	√

### 2.2 Competition in the dual-channel supply chain

Research on supply chains has been the focus of relevant scholars. These include supply chain risk management [32, 33], closed-loop supply chains [33–36], and dual-channel supply chains. However, this paper will focus on the dual-channel supply chain. There are many research points on the dual-channel supply chain. Among them, more attention is given to the horizontal competition of the two channels. Furthermore, competition between online and offline channels has always been the focus of scholars’ research. Particularly noteworthy are the “showroom” and “BOPS” issues. Most research has found that the “showroom” intensifies the competition between traditional channels and electronic channels and reduces the profit of traditional retailers and e-tailers [37]. However, although some studies find that BOPS may intensify online and offline competition, they also discover that BOPS (Buy-Online-and-Pick-Up-in-Store) revenue can be shared across channels to alleviate incentive conflicts [38]. In addition, Colombo and Matsushima [39] considered the spatial competition between offline and online retailers from the perspective of consumers’ online and offline purchase efficiency. Chai et al. [40] showed that the store brand strategy may be an effective means for offline stores to ease the competition of e-commerce in opening exhibition halls.

Regarding the competition between online platforms, the issue of platform competition brought about by different sales models has always been a hot topic in the supply chain field. For example, Abhishek et al. [41] studied the competition between two e-tailers from the perspective of the choice of reselling model and agency selling model. Recently, some scholars have discussed the competition between two online platforms based on the perspective of blockchain technology. Choi et al. [42] established a duopoly game model of two rental service platforms with the support of blockchain technology and analyse the impact of information disclosure brought about by blockchain technology on platform competition. Different from the study of Choi, Jiang et al. [43] established a game theory model to study how the blockchain platform’s decision on block size and transaction fees affects the competition between blockchain platforms. As shown in Table 2, we summarize the previous literature regarding the horizontal competition between two retailers in dual-channel supply chains.

**Table 2:** Summary of the related literature regarding the horizontal competition between two retailers in dual-channel supply chains.

Literature	Research Methodology	Competing members in the supply chain	Competition in dual-channel supply chains between retailers		
			No involves	Involves brand value	Involves blockchain
Moosavi et al. [32]	Literature Review				
Asghari [35]	Model analysis	Offline and offline			
Fathollahi et al. [34]	Design science				
Simonetto et al. [33]	Literature Review				
Berlin et al. [36]	Design science				
Balakrishnan et al. [37]	Model analysis	Online and offline	√		
Gao and Su [38]	Model analysis	Online and offline	√		
Colombo and Matsushima [39]	Model analysis	Online and offline	√		
Chai et al. [40]	Model analysis	Online and offline		√	
Abhishek et al. [41]	Model analysis	Online and online	√		
Choi et al. [42]	Model analysis	Online and online			√
Jiang et al. [43]	Model analysis	Online and online			√
Our study	Model analysis	Online and online		√	√

### 2.3 Heterogeneous consumers

Consumer heterogeneity has always been a key issue for scholars in various fields, and many scholars build models based on different consumer heterogeneous types. Most scholars regard product price and quality as the most important factors that directly affect demand. Therefore, they all use price and quality as indicators to distinguish consumer types to carry out research in the field of supply chain operations [44–48]. The focus of consumers’ attention on products is ever changing. In addition to product price and quality, there are also consumers who pay more attention to product guarantees and product valuations. Therefore, Lei et al. [49] explore the issue of companies’ dynamic pricing of products and warranty services based on the heterogeneity of consumers’ attitudes towards warranty. Zheng et al. [50] and Qiu et al. [51] and Wang et al. [52]conduct research by examining the role of consumer valuation heterogeneity in corporate profits. Regarding food and green energy supply chain management, some researchers innovatively study the pricing of genetically modified foods by considering the heterogeneity of consumers’ attitudes towards genetically modified foods [53, 54] study the implementation of green innovation in the entire supply chain by dividing heterogeneous consumers into two types: green and nongreen. Sharma et al. [55] consider heterogeneous consumer preferences for green and non-green luxury goods in a luxury context. Although there are related studies that use blockchain as a research background, the analysis is not based on consumers’ sensitivity to blockchain. For example, Li et al. [56] discuss the pricing strategy of the supply chain from the perspective of consumers’ heterogeneity of transaction costs in the context of blockchain. As shown in Table 3, we summarize the previous literature regarding the heterogeneity of consumers in supply chains.

**Table 3:** Summary of the related literature regarding the heterogeneity of consumers in supply chains.

Literature	Consumer heterogeneous type						
	Price or fee	Quality	Warranty	Genetic food	Green innovation	Product valuations	Blockchain
Shi et al. [44]		√					
Herbon [45]	√	√					
Kopczewski et al. [46]	√						
Homburg et al. [47]	√						
Lin et al. [48]	√		√				
Lei et al. [49]			√				
Zheng et al. [50]						√	
Qiu et al. [51]						√	
Wang et al. [52]						√	
Li and Basu [53]				√			
Meng et al. [54]					√		
Sharma et al. [55]					√		
Li et al. [56]	√						
Our study							√

### 2.4 Research gaps

By combing the above literature, we summarise the gaps between this paper and the relevant literature as follows:

First, the above studies are all theoretical and applied studies on today’s popular technology, blockchain. Most articles reveal that the adoption of blockchain technology plays a major role in enhancing the competitiveness of enterprises in various industries. Similar to the above studies, we also discuss the impact of blockchain technology on supply chain operations. However, many articles use qualitative methods to analyse the benefits of blockchain characteristics for a single-channel supply chain. While some articles have quantitatively studied the impact of blockchain technology on the profitability of supply chain members through a gaming approach, very few articles have studied the impact of blockchain technology on competition between two e-tailing platforms based on an e-commerce context. Therefore, based on the above studies, this paper focuses on competition between e-retailers in a dual-channel supply chain to further understand the value of blockchain adoption by discussing the relevance factors such as consumer sensitivity to blockchain, agency agreements, and brand value effects.

Second, the above articles have deeply studied the horizontal competition issues of dual-channel supply chain members. Similar to the above reviewed literature, this paper also focuses on horizontal competition between two retailers. However, the above literature studies fierce competition between online retailers and offline retailers. This paper mainly focuses on competition between two e-tailers. Although there are a few articles that explore the effect of brand value, these articles do not take blockchain technology into consideration. Moreover, they do not combine the blockchain with the brand value effect to study the dual-channel supply chain competition of two e-tailers. Therefore, this paper focuses on blockchain technology adoption decisions for competing platforms. Specifically, weighing the relationship between the brand spillover effects of blockchain technology and the cost of blockchain usage in an online dual-channel competitive environment is key to the analysis.

Third, the above studies are all based on the heterogeneity of consumers from different perspectives. Similar to the above reviewed literature, this paper also focuses on the heterogeneity of consumers. However, unlike all of them, this paper divides heterogeneous consumers into two types: blockchain-sensitive consumers and nonblockchain-sensitive consumers. Therefore, this paper builds a linear function model of demand based on the heterogeneity of consumer sensitivity to blockchain technology, which provides the research basis for this paper to conduct sensitivity analysis of relevant factors and profit comparison.

## 3. Model description and notations

To analyse the competition between electronic platforms from the perspective of the brand value effect brought by the traceability of the blockchain, we develop a dual-channel platform competition game-theoretic duopoly economic model based on the heterogeneity of consumers’ sensitivity to blockchain technology, in which the two electronic platforms compete in the scenarios of using blockchain technology (the new e-tailer) and not using blockchain technology (the traditional e-tailer). We compare the equilibrium solutions in the two scenarios and analyse the factors affecting the profit level of competitive e-tailers. Moreover, we provide numerical simulation to support our theoretical findings, enhance the practical implications, and provide management insights into electronic platform competition in the context of blockchain technology.

### 3.1 Notations and definitions

To present a clearer picture of the meaning of the relevant parameters, the parameters needed in the modelling process are explained below in Table 4.

**Table 4:** Parameter definition.

Parameters	Definition
*D* ^*B*^	Demand in electronic channel with blockchain
*D* ^*E*^	Demand in electronic channel without blockchain
*a*	The market size
*θ*	The market share of blockchain-sensitive consumer
*β*	Cross-effort elasticity coefficient of demand
*p*	E-tail price
*c*_*b*_	Cost in electronic channel with blockchain
*c*_*e*_	Cost in electronic channel without blockchain
*Α*	The cost sensitivity to effort
*γ*_*b*_	Brand value effect with blockchain
Decision variables	Definition
*W*	Wholesale price of *S*
*t*_*b*_	Effort with blockchain of *ER*_1_
*t*_*e*_	Effort without blockchain of *ER*_2_

### 3.2 Model description

Consider a duopoly case in which in the market, two groups of e-tailers—e-tail channel with blockchain called new e-tailer (i.e., *ER*_1_) and e-tail channel without blockchain called traditional e-tailer (i.e., *ER*_2_), offer the same type of product to the market by directly selling the product from platforms which wholesale at a price *w* from the same supplier (i.e., *s*). In this paper, not surprisingly, we consider a two-level supply chain system consisting of a supplier and two competing e-tailers. Suppliers and two groups of e-tailers are risk neutral and completely rational; therefore, they make decisions according to the principle of expected profit maximization.

**Fig 1:**
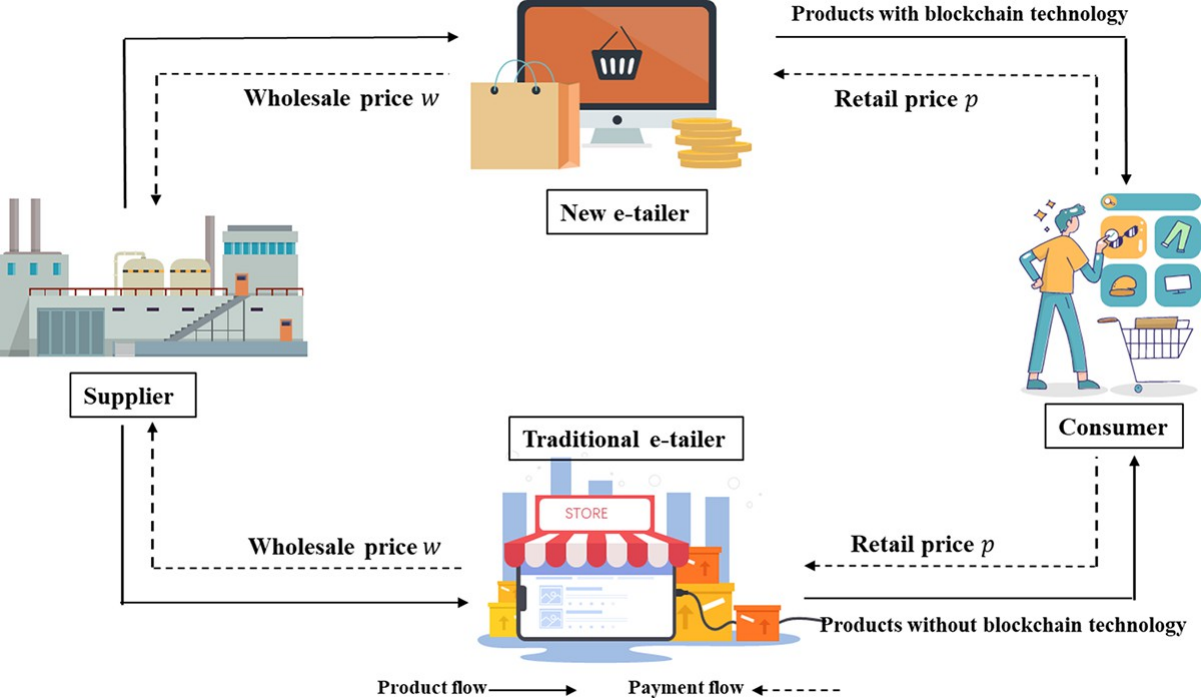
Supply chain structure under competition between the new e-tailer (blockchain technology-enabled) and the traditional e-tailer.

Some literature has classified heterogeneous consumers into two different types of consumers based on their different sensitivities to channels, and information sensitivities [44–47]. Similarly, we assume that there are two types of heterogeneous consumers in the market—a proportion of consumers *θ* prefer e-tail channels with blockchain (called “blockchain-sensitive”), and the rest 1‒*θ* are blockchain-insensitive. Based on interviews with VeChain and Houbi China, Pun et al. [57] found that these two blockchain providers use a variable-cost model, and there is no fixed cost for using their blockchain service. Thus, according to the Pun’s research [57], we assume that adopting blockchain technology incurs a unit variable-cost *c*_*b*_ = *αt*_*b*_ in the e-tail channel with blockchain. Similarly, a unit variable-cost *c*_*e*_ = *αt*_*e*_ for the e-tail channel without blockchain will be incurred, where cost is linearly related to the blockchain effort of *ER*_1_or the marketing effort of *ER*_2_ (i.e., *t*_*b*_ or *t*_*e*_) and hence increasing in *t*_*b*_ or *t*_*e*_. *α* (0<*α*<1) is the cost sensitivity to the effort. Meanwhile, trust in the product is improved, and consumers are more loyal to the product when using blockchain technology, thus bringing consumer stickiness (JD.com, 2020). We call it the brand value effect *γ*_*b*_ (*γ*_*b*_≥0) and assume it an exogenous variable of the profit of the blockchain channel; otherwise, the platform will not adopt blockchain technology. It is worth noting that, through relevant literature research, we find that consumers who pay attention to the trust of product and brand, price is not their primary consideration (Marks and Spencer Press Release, 2018). In this article, we focus on the impact of the e-tailer’s effort on whether it uses the blockchain (i.e., *t*_*b*_ and *t*_*e*_) to improve consumer trust and thus affect demand. Therefore, we do not consider the impact of price on demand and assume that two e-tailers set the same retail price. As this paper focuses on the brand spillovers and unit adoption costs associated with the adoption of blockchain technology. Therefore, to simplify the research, this paper does not consider the initial investment cost of the implementation of the blockchain. Thus, we assume that the investment cost of the blockchain is normalized to zero. The supply chain structure diagram under competition between the new (blockchain technology-enabled) e-tailer and the traditional e-tailer in this paper is shown in Fig 1.

Specifically, Fig 1 shows that a single manufacturer first sells its products to a new e-tailer and a traditional e-tailer at wholesale price *w* respectively. For a new e-tailer with blockchain technology, it first adopts blockchain technology for its wholesale products and then sells them to consumers at retail price *p*. For a traditional e-tailer that does not adopt blockchain technology, it sells the products directly to consumers at retail price *p*. For traditional e-tailers that do not adopt blockchain technology, they sell their products directly to consumers at retail price *p*. We use a solid line to show the direction of product flow, along with a dashed line for the direction of payment flow. Specifically, for the product flow, products first flow from the supplier to both the new e-tailer and the traditional e-tailer at the same time, and then products with blockchain technology and products without blockchain technology flow from the new e-tailer and the traditional e-tailer to consumers at the same time. For the payment flow, the new e-tailer and the traditional e-tailer first pay the wholesale price per unit of product, *w*, to the supplier, and then the consumer pays the retail price per unit of product, *p*, to both types of e-tailer at the same time as he or she purchases the product.

In this paper, the demand of the two electronic channels is determined by the market size *a*, the market share of consumers’ preference for e-tail channels with blockchain (called “blockchain-sensitive”) *θ*, the cross-effort elasticity coefficient *β* and the effort of *ER*_1_ or *ER*_2_ (i.e., *t*_*b*_ or *t*_*e*_). From the research of Cole R et al. [12], we know that the degree of consumer trust in the product is a key factor influencing the purchase decision. Thus, we take the platform’s efforts to gain consumer trust as a factor that directly affects channel demand regardless of price [42]. The modified demand functions of the two channels are shown as follows:

$$D^{B}=\theta a+t_{b}-\beta t_{e}$$ (1)

$$D^{E}=(1-\theta )a+t_{e}-\beta t_{b}$$ (2)

where superscript *B* or subscript *b* denotes the e-tail channel with blockchain, superscript *E* or subscript *e* denotes the e-tail channel without blockchain and *D* is the basic product demand. *θ*, which reflects consumers’ preference for e-tail channels with blockchain. Conversely, 1‒*θ* reflects consumers’ preference for e-tail channels without blockchain. *β* is the cross-effort elasticity coefficient between the product effort and demand for an alternative product. Meanwhile, the channel demand is directly proportional to the channel’s own effort and inversely proportional to the effort of the other channel. For analytical convenience, we assume that the effort elasticity coefficient of self-effort is equal to 1, and the demand is impacted by the product effort of another channel less than it is by its channel effort (i.e., 0<*β*<1).

## 4. Equilibria analysis

In this section, we present the equilibria for *S*, *ER*_1_ and *ER*_2_, which are all played at Stackelberg with the supplier being the leader. Therefore, the supplier informs the chain to produce and distribute a certain product and sell at a wholesale price *w*. In channel *B* (i.e., e-tail channel with blockchain), firm *ER*_1_ seeks to carry out the supply chain on a blockchain. Thus, firm *ER*_1_ needs to make effort *t*_*b*_. Meanwhile, in channel *E* (i.e., e-tail channel without blockchain), although firm *ER*_2_ adopts a traditional online platform for supply chain negotiations rather than using blockchain technology, there are still other efforts *t*_*e*_ to pay. Finally, supply chain transactions occur on platforms, and the games evolve according to the following steps: The supplier will be able to deliver products at a wholesale price *w*; the two e-tailers react to this determine by setting the same retail price *p*, which is exogenous, and making optimal effort *t*_*b*_ and *t*_*e*_.

Based on the above, the profit functions (Π_*S*_, $\Pi_{ER_{1}}^{B}$ and $\Pi_{ER_{2}}^{E}$) of the supplier and two e-tailers are given by Eqs (3), (4) and (5), respectively:

$$\Pi_{S}=(w-\alpha t_{b})D^{B}+\gamma_{b}+wD^{E}$$ (3)

$$\Pi_{ER_{1}}^{B}=(p-w-\alpha t_{b})D^{B}+\gamma_{b}$$ (4)

$$\Pi_{ER_{2}}^{E}=(p-w-\alpha t_{e})D^{E}$$ (5)

It is worth noting that the supplier’s profit includes two major parts: the profit obtained from the blockchain channel and the profit obtained from the traditional channel without blockchain technology. When the e-tailer implements blockchain technology in the electronic channel, the upstream supplier must also join it. Therefore, the operating cost and the brand value effect of implementing the blockchain exist in the supplier’s profit function at the same time. However, in the traditional channel without blockchain technology, since channel operations are mainly implemented by e-tailers, the supplier’s operating costs are not considered.

To obtain the optimal decision, we substitute (1), (2) into (3), (4), and (5); the second derivative of *t*_*b*_ in (4) and the second derivative of *t*_*e*_ in (5) are less than zero. Let the first derivative of *t*_*b*_ and the first derivative of *t*_*e*_ equal zero; then, we obtain $t_{b}^{*}$ and $t_{e}^{*}$. Next, substituting $t_{b}^{*}$ and $t_{e}^{*}$ into (3), finding the first derivative of *w* and letting it equal zero, we can obtain the optimal wholesale price w*. The equilibria of the game are displayed in the following theorem:

**Theorem 1** The optimal firms’ strategies in the game are:

$$w^{*}=\frac{a(4+\beta \theta (2-\beta ))\alpha +2p(1-\beta )(3-\beta )(\beta +2)}{2(1-\beta )(5-2\beta )(\beta +2)}$$ (6)

$$t_{b}^{*}=\frac{a(-(5-4\beta )(2-\beta )\theta +4\beta^{2}-6\beta -2)\alpha +2p(\beta +2)(1-\beta )}{2\alpha (\beta +2)(1-\beta )(5-2\beta )}$$ (7)

$$t_{e}^{*}=\frac{a((4\beta^{2}-15\beta +10)\theta -4(3-2\beta ))\alpha +2p(\beta +2)(1-\beta )}{2\alpha (\beta +2)(1-\beta )(5-2\beta )}$$ (8)

Furthermore, substituting Eqs (6), (7) and (8) into Eqs (3), (4) and (5), the optimal profits of the supplier and two e-tailers can be obtained as follows:

$$\begin{array}{l} \Pi_{S}^{*}=\frac{1}{4\alpha {\left(\beta +2\right)}^{2}(5-2\beta )(1-\beta )}(a^{2}((10-9\beta )(2-\beta )\theta^{2}+8\beta (3-2\beta )\theta +8\beta^{2}+4\beta +4)\alpha^{2}\\ +(a(-4\beta (\beta +2)(1-\beta )\theta +4(2\beta +3)(\beta +2)(1-\beta ))p-4\gamma_{b}{\left(\beta +2\right)}^{2}(2\beta -5)(1-\beta ))\alpha \\ +4p^{2}{\left(\beta +2\right)}^{2}{\left(1-\beta \right)}^{2}) \end{array}$$ (9)

$$\begin{array}{l} \Pi_{ER_{1}}^{B*}=\frac{1}{4\alpha {\left(5-2\beta \right)}^{2}{\left(\beta +2\right)}^{2}}(a^{2}(25{\left(2-\beta \right)}^{2}\theta^{2}+20(2\beta -1)(2-\beta )\theta +4{\left(2\beta -1\right)}^{2})\alpha^{2}\\ +(2p(1-\beta )(2-\beta )(2+\beta )\theta +8p(1-\beta )(2\beta -1)(\beta +2))a+4\gamma_{b}{\left(\beta +2\right)}^{2}{\left(5-2\beta \right)}^{2})\alpha \\ +4p^{2}{\left(\beta +2\right)}^{2}{\left(1-\beta \right)}^{2}) \end{array}$$ (10)

$$\Pi_{ER_{2}}^{E*}=\frac{{\left(a(3\beta \theta -10\theta +8)\alpha +2p(\beta +2)(1-\beta )\right)}^{2}}{4\alpha {\left(5-2\beta \right)}^{2}{\left(\beta +2\right)}^{2}}$$ (11)

Theorem 1 shows that both the decision variables (i.e., w*, $t_{b}^{*}$ and $t_{e}^{*}$) and the profit level of the supplier and the two e-tailers (i.e., $\Pi_{S}^{*}$, $\Pi_{ER_{1}}^{B*}$ and $\Pi_{ER_{2}}^{E*}$) are affected by parameters *α*, *β* and *θ*. In addition, the profit level of the supplier and the e-tailer using blockchain technology (i.e., $\Pi_{S}^{*}$ and $\Pi_{ER_{1}}^{B*}$) are also affected by the brand value effect at the same time.

To further understand how parameters (*α*, *β*, *θ*) affect decision variables (i.e., w*, $t_{b}^{*}$ and $t_{e}^{*}$) and the profit level of the supplier and the two e-tailers (i.e., $\Pi_{S}^{*}$, $\Pi_{ER_{1}}^{B*}$ and $\Pi_{ER_{2}}^{E*}$), we will conduct a specific analysis of the properties of decision variables and profits.

### 4.1 The property analysis for the decision variables

#### 4.1.1 The property analysis for the optimal *W*. Property 1

With the ascension of *α*, *β*, and *θ*, the optimal wholesale price will increase. Specifically, three cases are possible:

(i) $\frac{\partial w^{*}}{\partial \alpha }>0$; (ii) $\frac{\partial w^{*}}{\partial \beta }>0$; (iii) $\frac{\partial w^{*}}{\partial \theta }>0$.

Property 1 shows that the optimal wholesale prices always increase with increasing cost sensitivity to effort, the cross-effort elasticity coefficient of demand or the market share of blockchain-sensitive consumers.

The reason for this result may be that when the cost sensitivity to effort increases, the costs of the electronic platform will increase more with increasing effort. To obtain more profits, e-tailers can only further increase prices. The supplier predicts that e-tailers’ prices will rise, so the supplier increases wholesale prices. Demand may decrease when the cross-effort elasticity coefficient of demand increases; thus, the supplier will increase wholesale prices to maintain a higher level of profits. E-tailers tend to implement blockchain technology when the market share of blockchain-sensitive consumers increases, and the supplier further increases wholesale prices to obtain more profits in this situation.

#### 4.1.2 The property analysis for the optimal *t*_*b*_. Property 2

With the ascension of *α* and *θ*, the optimal effort of *ER*_1_ will decrease. However, the influence of *β* on the optimal effort of *ER*_1_ depends on the value range of *α*. Specifically, three cases are possible:

(i) $\frac{\partial {t_{b}}^{*}}{\partial \alpha }<0$; (ii) If $0<\alpha <-\frac{B_{1}}{A_{1}}$, therefore, $\frac{\partial {t_{b}}^{*}}{\partial \beta }>0$; If $-\frac{B_{1}}{A_{1}}\leq \alpha <1$, therefore, $\frac{\partial {t_{b}}^{*}}{\partial \beta }\leq 0$; (iii) $\frac{\partial {t_{b}}^{*}}{\partial \theta }<0$.

Property 2 shows that the effort with blockchain of *ER*_1_ always decreases with the cost sensitivity to effort or the market share of blockchain-sensitive consumer increases. However, when the cost sensitivity to effort is small, the effort with blockchain of *ER*_1_ always increases as the cross-effort elasticity coefficient of demand increases; when the cost sensitivity to effort is large, the effort with blockchain of *ER*_1_ will decrease as the cross-effort elasticity coefficient of demand increases.

The reason for this result may be that when the cost sensitivity to effort increases, the cost of the electronic platform will increase more with the increase of effort, and the e-tailer will reduce effort to implement blockchain technology to lower the cost as much as possible. When the cost sensitivity to effort is small, the cost of the electronic platform will increase less with the increase of effort, so the e-tailer is willing to make greater effort in blockchain technology. Therefore, the e-tailer is still willing to make greater effort in blockchain technology even if as the cross-effort elasticity coefficient of demand increases; in contrast, when the cost sensitivity to effort is greater, the cost of the electronic platform will increase more with the increase of effort. In this case, the e-tailer will reduce effort to implement blockchain technology as the cross-effort elasticity coefficient of demand increases considering the larger costs. However, when the market share of blockchain-sensitive consumers increases, the e-tailer may reduce effort to implement blockchain technology because of the larger consumer’s market share.

#### 4.1.3 The property analysis for the optimal *t*_*e*_. Property 3

With the ascension of *α*, the optimal effort of *ER*_2_ will decrease. However, the influence of *β* on the optimal effort of *ER*_2_ depends on the value range of *α*, and the influence of *θ* on the optimal effort of *ER*_2_ depends on the value range of *β*. Specifically, three cases are possible:

(i) $\frac{\partial {t_{e}}^{*}}{\partial \alpha }<0$;

(ii) If $0<\alpha <-\frac{B_{2}}{A_{2}}$, therefore, $\frac{\partial {t_{e}}^{*}}{\partial \beta }>0$; If $-\frac{B_{2}}{A_{2}}\leq \alpha <1$, therefore, $\frac{\partial {t_{e}}^{*}}{\partial \beta }\leq 0$;

*(iii) If* 0<*β*<0.8672*, therefore, $\frac{\partial {t_{e}}^{*}}{\partial \theta }>0$; if* 0.8672≤*β*<1*, therefore, $\frac{\partial {t_{e}}^{*}}{\partial \theta }\leq 0$.*

Property 3 shows that the effort without blockchain of *ER*_2_ always decreases as the cost sensitivity to effort increases. When the cost sensitivity to effort is small, the effort without blockchain of *ER*_2_ always increases as the cross-effort elasticity coefficient of demand increases; when the cost sensitivity to effort is large, the effort without blockchain of *ER*_2_ will decrease as the cross-effort elasticity coefficient of demand increases. The above properties are the same as property 2; the only difference is that when the cross-effort elasticity coefficient of demand is large enough, the effort without blockchain of *ER*_2_ always decreases as the market share of blockchain-sensitive consumers increases; when the cross-effort elasticity coefficient of demand is not large enough, the effort without blockchain of *ER*_2_ always increases as the market share of blockchain-sensitive consumers increases.

The reason for this result may be that when the cost sensitivity to effort increases, the cost of the electronic platform will increase more as the effort increases, and the traditional e-tailer will reduce effort to lower costs as much as possible. When the cost sensitivity to effort is small, the cost of the electronic platform will increase less with the increases of effort, the traditional e-tailer is willing to make greater effort to sell better products, so even if the cross-effort elasticity coefficient of demand increases, the traditional e-tailer is still willing to make greater effort to sell better products; in contrast, when the cost sensitivity to effort is greater, the cost of electronic platform will increase more with the increase of effort. Under this circumstance, the traditional e-tailer will reduce effort as the cross-effort elasticity coefficient of demand increases when considering larger costs. When the cross-effort elasticity coefficient of demand is large enough, it means that effort to implement blockchain has a greater impact on the demand of this channel. Under this circumstance, as the proportion of blockchain-sensitive consumers increases, it is difficult for the traditional e-tailer to obtain more demand even if effort increases, so the traditional e-tailer decides not to implement effort; when the cross-effort elasticity coefficient of demand is not large enough, it means that effort to implement blockchain does not have a great impact on the demand of this channel. In this case, as the proportion of blockchain-sensitive consumers increases, the traditional e-tailer decides to increase effort to obtain more demand.

To show the influence of parameters (*α*, *β*, *θ*) on decision variables (w*, $t_{b}^{*}$ and $t_{e}^{*}$) more clearly, we use the following table to analyse sensitivity. Table 5 summarizes the results.

**Table 5:** Sensitivity analysis towards the critical parameters *α*, *β*, and *θ*.

	*α*	*β*	*θ*
w*↑	↑	↑	↑
$t_{b}^{*}↑$	↓	$↑(0<\alpha <-\frac{B_{1}}{A_{1}})$ $↓(-\frac{B_{1}}{A_{1}}\leq \alpha <1)$	↓
$t_{e}^{*}↑$	↓	$↑(0<\alpha <-\frac{B_{2}}{A_{2}})$ $↓(-\frac{B_{2}}{A_{2}}\leq \alpha <1)$	↑(0<*β*<0.8672) ↓(0.8672 ≤*β*<1)

### 4.2 The property analysis for the optimal profits

#### 4.2.1 The property analysis for the optimal Π_*S*_. Property 4

The influence of *α*, *β*, and *θ* on the optimal profit of the supplier depends on the value range of *α*. Specifically, three cases are possible:

(i) If $0<\alpha <\sqrt{-\frac{B_{3}}{A_{3}}}$, therefore, $\frac{\partial \Pi_{S}^{*}}{\partial \alpha }<0$; If $\sqrt{-\frac{B_{3}}{A_{3}}}\leq \alpha <1$, therefore, $\frac{\partial \Pi_{S}^{*}}{\partial \alpha }\geq 0$;

(ii) If $0<\alpha <\frac{-B_{4}+\sqrt{{\left(B_{4}\right)}^{2}-4A_{4}C_{4}}}{2A_{4}}$, therefore, $\frac{\partial \Pi_{S}^{*}}{\partial \beta }<0$;

If $\frac{-B_{4}+\sqrt{{\left(B_{4}\right)}^{2}-4A_{4}C_{4}}}{2A_{4}}\leq \alpha <1$, therefore, $\frac{\partial \Pi_{S}^{*}}{\partial \beta }\geq 0$;

(iii) If $0<\alpha <-\frac{B_{5}}{A_{5}}$, therefore, $\frac{\partial \Pi_{S}^{*}}{\partial \theta }<0$; if $-\frac{B_{5}}{A_{5}}\leq \alpha <1$, therefore, $\frac{\partial \Pi_{S}^{*}}{\partial \theta }\geq 0$.

Property 4 shows that when the cost sensitivity to effort is in a small range, the supplier’s optimal profit level will decrease with the cost sensitivity to effort, and the cross-effort elasticity coefficient of demand and the market share of blockchain-sensitive consumers increases. However, when the cost sensitivity to effort is in a larger range, the supplier’s profit level will increase as the cost sensitivity to effort, the cross-effort elasticity coefficient of demand and the market share of blockchain-sensitive consumers increase. Property 4 further illustrates that the important factor that affects the supplier’s profit level is cost sensitivity to effort.

The reason for this result may be that when the cost sensitivity to effort increases in a smaller range, the cost of the electronic platform will increase with increasing effort. Under this circumstance, the e-tailer’s costs gradually rise, resulting in a decrease in the profit level, which in turn leads to a decrease in the profit level of the supplier. However, when the cost sensitivity to effort increases in a larger range, because the cost of the electronic platform is too high, the e-tailer will reduce effort instead to lower the cost of the platform effort. Therefore, the profit level of the supplier increases.

#### 4.2.2 The property analysis for the optimal $\Pi_{ER_{1}}^{B}$. Property 5

The influence of *α* and *β* on the optimal profit of *ER*_1_ depends on the value range of *α*. However, with the ascension of *θ*, the optimal profit of *ER*_1_ will increase. Specifically, three cases are possible:

(i) If $0<\alpha <-\frac{B_{6}}{A_{6}}$, therefore, $\frac{\partial \Pi_{ER_{1}}^{B*}}{\partial \alpha }<0$; If $-\frac{B_{6}}{A_{6}}\leq \alpha <1$, therefore, $\frac{\partial \Pi_{ER_{1}}^{B*}}{\partial \alpha }\geq 0$;

(ii) If $0<\alpha <-\frac{B_{8}}{A_{8}}$, therefore, $\frac{\partial \Pi_{ER_{1}}^{B*}}{\partial \beta }<0$; If $-\frac{B_{8}}{A_{8}}\leq \alpha <1$, therefore, $\frac{\partial \Pi_{ER_{1}}^{B*}}{\partial \beta }\geq 0$;

(iii) $\frac{\partial \Pi_{ER_{1}}^{B*}}{\partial \theta }>0$.

Property 5 shows that when the cost sensitivity to effort is in a small range, the optimal profit level of the e-tailer implementing blockchain technology will decrease with the cost sensitivity to effort or the cross-effort elasticity coefficient of demand increases. However, when the cost sensitivity to effort is in a larger range, the e-tailer’s profit level will increase as the cost sensitivity to effort or the cross-effort elasticity coefficient of demand increases. The reason for this result may be that when the cost sensitivity to effort increases in a smaller range, the cost of the electronic platform will increase with increasing effort. Under this circumstance, the e-tailer’s cost gradually increases, resulting in a decrease in profit. However, when the cost sensitivity to effort is increased in a larger range, because the effort cost of the electronic platform is too high, the e-tailer will reduce the effort to lower the effort cost of the platform. Therefore, the e-tailer’s profit level increases. This is similar to property 4.

Unlike property 4, regardless of the cost sensitivity to effort, the optimal profit level of an e-tailer implementing blockchain technology will always increase as the market share of blockchain-sensitive consumers increases. The reason for this result may be that there are more blockchain consumers in the market when the proportion of the blockchain-sensitive consumer market increases, and the e-tailer can gain more market share when implementing blockchain technology, therefore bringing the rise of profits.

#### 4.2.3 The property analysis for the optimal $\Pi_{ER_{2}}^{E}$. Property 6

Similar to property 5, the influence of *α* and *β* on the optimal profit of *ER*_2_ depends on the value range of *α*. In contrast to property 5, with the ascension of *θ*, the optimal profit of *ER*_2_ will decrease. Specifically, three cases are possible:

(i) If $0<\alpha <-\frac{B_{11}}{A_{11}}$, therefore, $\frac{\partial \Pi_{ER_{2}}^{E*}}{\partial \alpha }<0$; If $-\frac{B_{11}}{A_{11}}\leq \alpha <1$, therefore, $\frac{\partial \Pi_{ER_{2}}^{E*}}{\partial \alpha }\geq 0$;

(ii) If $0<\alpha <-\frac{B_{13}}{A_{13}}$, therefore, $\frac{\partial \Pi_{ER_{2}}^{E*}}{\partial \beta }<0$; If $-\frac{B_{13}}{A_{13}}\leq \alpha <1$, therefore, $\frac{\partial \Pi_{ER_{2}}^{E*}}{\partial \beta }\geq 0$;

(iii) $\frac{\partial \Pi_{ER_{2}}^{E*}}{\partial \theta }<0$.

Property 6 shows that when the cost sensitivity to effort is in a small range, the optimal profit level of the traditional e-tailer will decrease with the cost sensitivity to effort or the cross-effort elasticity coefficient of demand increases. When the cost sensitivity to effort is in a larger range, the profit level of the traditional e-tailer will increase as the cost sensitivity to effort or the cross-effort elasticity coefficient of demand increase. The reason for this result may be that when the cost sensitivity to effort increases in a smaller range, the cost of the electronic platform will increase with increasing effort. For this time, the cost of the traditional e-tailer will gradually increase, resulting in a decrease in profit; when the cost sensitivity to effort increases on a larger scale, the traditional e-tailer will reduce effort to reduce the cost of effort of the platform due to the high cost of effort of the electronic platform. Therefore, the profit level of the traditional e-tailer increases. This is similar to property 5.

Different from property 5, regardless of the cost sensitivity to effort, the optimal profit level of traditional e-tailers always increases as the market share of blockchain-sensitive consumers increases. The reason for this result may be that there are more blockchain consumers in the market when the proportion of the blockchain-sensitive consumer market increases, and the traditional (not implementing blockchain technology) e-tailer can only obtain a smaller market share. This leads to a reduction in the level of profit.

To show the influence of parameters (*α*, *β*, *θ*)on the optimal profits ($\Pi_{S}^{*}$, $\Pi_{ER_{1}}^{B*}$and $\Pi_{ER_{2}}^{E*}$) more clearly, we use the following table to analyse the sensitivity. Table 6 summarizes the results.

**Table 6:** Sensitivity analysis towards the critical parameters *α*, *β*, and *θ*.

	*α*	*β*	*θ*
$\Pi_{S}^{*}↑$	$↓(0<\alpha <\sqrt{-\frac{B_{3}}{A_{3}}})$ $↑(\sqrt{-\frac{B_{3}}{A_{3}}}\leq \alpha <1)$	$↓(0<\alpha <\frac{-B_{4}+\sqrt{\Delta_{4}}}{2A_{4}})$ $↑(\frac{-B_{4}+\sqrt{\Delta_{4}}}{2A_{4}}\leq \alpha <1)$	$↓(0<\alpha <\sqrt{-\frac{B_{5}}{A_{5}}})$ $↑(\sqrt{-\frac{B_{5}}{A_{5}}}\leq \alpha <1)$
$\Pi_{ER_{1}}^{B*}↑$	$↓(0<\alpha <-\frac{B_{6}}{A_{6}})$ $↑(-\frac{B_{6}}{A_{6}}\leq \alpha <1)$	$↓(0<\alpha <-\frac{B_{8}}{A_{8}})$ $↑(-\frac{B_{8}}{A_{8}}\leq \alpha <1)$	↑
$\Pi_{ER_{2}}^{E*}↑$	$↓(0<\alpha <-\frac{B_{11}}{A_{11}})$ $↑(-\frac{B_{11}}{A_{11}}\leq \alpha <1)$	$↓(0<\alpha <-\frac{B_{13}}{A_{13}})$ $↑(-\frac{B_{13}}{A_{13}}\leq \alpha <1)$	↓

### 4.3 Comparison analysis

#### 4.3.1 The comparison analysis for the optimal *t*_*b*_ and *t*_*e*_. Property 7

The comparison results between the optimal effort of *ER*_1_ and *ER*_2_ depend on the value range of *θ*. Specifically, two cases are possible:

(i) If $0<\theta <\frac{1}{2}$, therefore, $t_{b}^{*}>t_{e}^{*}$; (ii) If $\frac{1}{2}\leq \theta <1$, therefore, $t_{b}^{*}\leq t_{e}^{*}$.

Property 7 shows that when the blockchain-sensitive consumer market accounts for less than half of the market share, the effort of the new e-tailer who implements blockchain technology will be greater than the effort of the traditional e-tailer who does not implement blockchain technology; in contrast, when the blockchain-sensitive consumer market accounts for more than half of the market share, the effort of the new e-tailer who implements blockchain technology will be less than the effort of the traditional e-tailer who does not implement blockchain technology. The reason for this result may be that when the blockchain-sensitive consumer market accounts for less than half of the market share, the new e-tailer implementing blockchain technology wants to work harder to gain a larger share of the consumer market. In contrast, less effort is required.

#### 4.3.2 The comparison analysis for the optimal $\Pi_{ER_{1}}^{B}$ and $\Pi_{ER_{2}}^{E}$. Property 8

The comparison results between the optimal profit of *ER*_1_ and *ER*_2_ depend on the value range of *α*, *θ*, and *γ*_*b*_. Specifically, three cases are possible:

(i) If $0<\theta <\frac{1}{2}$ and $0<\gamma_{b}<\frac{2ap(1-\beta )(1-2\theta )}{\left(5-2\beta )(\beta +2\right)}$, therefore, $\Pi_{ER_{1}}^{B*}<\Pi_{ER_{2}}^{E*}$;

(ii) If $0<\theta <\frac{1}{2}$ and $\gamma_{b}\geq \frac{2ap(1-\beta )(1-2\theta )}{\left(5-2\beta )(\beta +2\right)}$:

when $0<\alpha <-\frac{B_{16}}{A_{16}}$, therefore, $\Pi_{ER_{1}}^{B*}>\Pi_{ER_{2}}^{E*}$; when $-\frac{B_{16}}{A_{16}}\leq \alpha <1$, $\Pi_{ER_{1}}^{B*}\leq \Pi_{ER_{2}}^{E*}$;

(iii) If $\frac{1}{2}\leq \theta <1$, therefore, $\Pi_{ER_{1}}^{B*}\geq \Pi_{ER_{2}}^{E*}$.

where $A_{16}=a^{2}(-2\beta \theta^{2}+(5\beta +6)\theta -2\beta -3)$, $B_{16}=2ap(\beta +2)(1-\beta )(2\theta -1)+\gamma_{b}(5-2\beta ){\left(\beta +2\right)}^{2}$. Property 8 shows that when the market share of blockchain-sensitive consumers and the brand value effect with blockchain are both in a small range, the profit level of the new e-tailer who implements blockchain technology is less than that of the traditional e-tailer who does not implement blockchain technology. When the market share of blockchain-sensitive consumers is relatively small but the brand value effect with blockchain is large, if the cost sensitivity to effort is in a small range, the profit level of the new e-tailer is greater than that of the traditional e-tailer that does not implement blockchain technology; conversely, if the cost sensitivity to effort is in a larger range, the profit level of the new e-tailer that implements blockchain technology is less than that of nonimplementation areas. When the blockchain-sensitive consumer market accounts for a relatively large market, the profit level of the new e-tailer that implements blockchain technology is always greater than that of the traditional e-tailer that does not implement blockchain technology.

## 5. Extended models

In the basic model, we consider a fixed-price wholesale model in which the supplier first simply sells their products to e-platforms, and then e-tailers sell their products to consumers on the platforms. However, the supplier will enter into agency selling agreements with e-platforms in many cases. Under this scenario, the supplier is required to share a percentage of the revenue as an agency fee for selling their products on the e-platform. How will such agency selling arrangements affect the e-tailers’ optimal effort and profit in the context of blockchain? To address these issues, we further explore them by building the following model.

In this scenario, the supplier enters into an agency agreement with two-sided e-tailers. The supplier can sell products directly to consumers through the blockchain e-platform and the traditional e-platform and share revenue with two-sided e-tailers as platform agency fees.

Based on this scenario, the profit functions ($\Pi_{S}^{A}$, $\Pi_{ER_{1}}^{A}$ and $\Pi_{ER_{2}}^{A}$) of the supplier and two e-tailers are given by Eqs (12), (13) and (14), respectively:

$$\Pi_{S}^{A}=((1-\xi_{1})p_{S}-\alpha t_{b}^{A})D^{B}+\gamma_{b}+(1-\xi_{2})p_{S}D^{E}$$ (12)

$$\Pi_{ER_{1}}^{A}=(\xi_{1}p_{S}-\alpha t_{b}^{A})D^{B}+\gamma_{b}$$ (13)

$$\Pi_{ER_{2}}^{A}=(\xi_{2}p_{S}-\alpha t_{e}^{A})D^{E}$$ (14)

where 0<*ξ*_1_<1 and 0<*ξ*_2_<1 represent the percentage of revenue shared by the supplier to the new e-tailer and traditional e-tailer, respectively. Not surprisingly, 0<1‒*ξ*_1_<1 and 0<1‒*ξ*_2_<1 represent the remaining revenues that the supplier keeps after sharing the needed proportions. Therefore, we have Theorem 2.

**Theorem 2** The optimal firms’ strategies in the game are:

$$t_{b}^{A*}=\frac{-a((2-\beta )\theta +\beta )\alpha +(2\xi_{1}+\beta \xi_{2})p_{S}}{\alpha (\beta +2)(2-\beta )}$$ (15)

$$t_{e}^{A*}=\frac{-a(\beta \theta +2(1-\theta ))\alpha +(\beta \xi_{1}+2\xi_{2})p_{S}}{\alpha (\beta +2)(2-\beta )}$$ (16)

Furthermore, substituting Eqs (15) and (16) into Eqs (12), (13) and (14), the optimal profits of the supplier and two e-tailers can be obtained as follows:

$$\Pi_{S}^{A*}=\frac{A_{17}\alpha^{2}+B_{17}\alpha +C_{17}}{\alpha {\left(\beta +2\right)}^{2}{\left(2-\beta \right)}^{2}}$$ (17)

$$\Pi_{ER_{1}}^{A*}=\frac{A_{18}\alpha^{2}+B_{18}\alpha +C_{18}}{\alpha {\left(\beta +2\right)}^{2}{\left(2-\beta \right)}^{2}}$$ (18)

$$\Pi_{ER_{2}}^{A*}=\frac{\left(a(\beta \theta +2(1-\theta )\alpha -p_{S}(\beta^{2}\xi_{2}+\beta \xi_{1}-2\xi_{2}\right)}{\alpha {\left(\beta +2\right)}^{2}{\left(2-\beta \right)}^{2}}$$ (19)

where *A*_17_ = *a*^2^(*βθ*‒*β*‒2*θ*)^2^,

$$B_{17}=\beta^{4}\gamma_{b}-ap_{S}(1-\theta \xi_{2})\beta^{3}-2(ap_{S}+4\gamma_{b})\beta^{2}+4ap_{S}(1-2\theta \xi_{2}-(1-\theta )\xi_{1})\beta -8a((1-\theta )\xi_{2}+\theta \xi_{1}-1)p_{S}+16\gamma_{b}$$

$$\begin{array}{l} C_{17}=-p_{S}^{2}((\xi_{1}^{2}+\xi_{2}^{2}-\xi_{1}-\xi_{2})\beta^{4}+(\xi_{1}\xi_{2}-\xi_{1}-\xi_{2})\beta^{3}+(-8\xi_{1}^{2}-7\xi_{2}^{2}+6\xi_{1}+6\xi_{2})\beta^{2}\\ +(-8\xi_{1}\xi_{2}+4\xi_{1}+4\xi_{2})\beta +12\xi_{1}^{2}+8\xi_{2}^{2}-8\xi_{1}-8\xi_{2}) \end{array};$$

$$A_{18}-A_{17},$$

$$B_{18}=\gamma_{b}\beta^{4}-2ap_{S}\xi_{1}(1-\theta )\beta^{3}-(2ap_{S}(2\theta \xi_{1}+(1-\theta )\xi_{2})+8\gamma_{b})\beta^{2}+4ap_{S}((1-\theta )\xi_{1}-\theta \xi_{2})\beta +8(a\theta \xi_{1}p_{S}+2\gamma_{b})$$

$$C_{18}=p_{S}^{2}{\left(\beta^{2}\xi_{1}+\beta \xi_{2}-2\xi_{1}\right)}^{2}.$$

Through the property of the previous basic model, we find that whether it is the supplier or the two e-tailers, the key influence on the profitability of the supply chain members is ultimately the cost sensitivity to effort *α*. Therefore, to simplify our study and strengthen the core results of this paper, we will further conduct a specific analysis of how parameters *α* affect decision variables (i.e., $t_{b}^{A*}$ and $t_{e}^{A*}$) and the profit level of the supply chain members (i.e., $\Pi_{S}^{A*}$, $\Pi_{ER_{1}}^{A*}$ and $\Pi_{ER_{2}}^{A*}$).

### 5.1 The property analysis for the decision variables

#### 5.1.1 The property analysis for the optimal $t_{b}^{A}$ and $t_{e}^{A}$. Property 9

With the ascension of *α*, the optimal effort of *ER*_1_ and *ER*_2_ will decrease. Specifically, two cases are possible:

(i) $\frac{\partial {t_{b}}^{A*}}{\partial \alpha }<0$; (ii) $\frac{\partial {t_{e}}^{A*}}{\partial \alpha }<0$. Similar to properties 2 and 3, property 9 still shows our core results. This implies that under the agency scenario, the effort with blockchain of *ER*_1_ always decreases with the cost sensitivity to effort or the market share of blockchain-sensitive consumers increases. Not surprisingly, the effort without blockchain of *ER*_2_ is negatively correlated with the cost sensitivity to effort.

**Corollary 1.** *(i) $\frac{\partial {t_{b}}^{A*}}{\partial \alpha }<\frac{\partial {t_{b}}^{*}}{\partial \alpha }$; (ii) $\frac{\partial {t_{e}}^{A*}}{\partial \alpha }<\frac{\partial {t_{e}}^{*}}{\partial \alpha }$.*

It is worth noting that Corollary 1 is also a very strong result. By comparing the agency scenario with the basic scenario, we find that cost sensitivity to effort has a greater impact on the e-tailer’s effort in the agency scenario. This is an interesting finding and reveals that e-tailers in the agency scenario may be more affected by costs due to the level of profit they receive.

### 5.2 The property analysis for the optimal profits

#### 5.2.1 The property analysis for the optimal $\Pi_{S}^{A}$. Property 10

The influence of *α* on the optimal profit of the supplier depends on the value range of *α*. Specifically, two cases are possible:

(i) If $0<\alpha <\sqrt{-\frac{B_{19}}{A_{19}}}$, therefore, $\frac{\partial \Pi_{S}^{A*}}{\partial \alpha }<0$; (ii) If $\sqrt{-\frac{B_{19}}{A_{19}}}\leq \alpha <1$, therefore, $\frac{\partial \Pi_{S}^{A*}}{\partial \alpha }\geq 0$.

Similar to property 4, property 10 still shows our core results. This implies that under the agency scenario, when the cost sensitivity to effort is in a smaller range, the supplier’s optimal profit level will decrease with the cost sensitivity to effort. Conversely, when the cost sensitivity to effort is in a larger range, the supplier’s optimal profit is positively correlated with the cost sensitivity to effort.

Corollary 2.

(i) If $0<\alpha <\min(\sqrt{-\frac{B_{3}}{A_{3}}},\sqrt{-\frac{B_{19}}{A_{19}}})$, therefore, $\frac{\partial \Pi_{S}^{A*}}{\partial \alpha }>\frac{\partial \Pi_{S}^{*}}{\partial \alpha }$;

(ii) If $\max(\sqrt{-\frac{B_{3}}{A_{3}}},\sqrt{-\frac{B_{19}}{A_{19}}})\leq \alpha <1$, therefore, $\frac{\partial \Pi_{S}^{A*}}{\partial \alpha }\leq \frac{\partial \Pi_{S}^{*}}{\partial \alpha }$. Corollary 2 is also a very strong result. By comparing the agency scenario with the basic scenario, we find that when the cost sensitivity to effort is in a smaller range, the profit of the supplier in the agency scenario decreases less than the profit of the supplier in the basic scenario for every unit increase in the cost sensitivity to effort. Namely, cost sensitivity to effort has less impact on the supplier’s profit in the agency scenario. However, when the cost sensitivity to effort is in a larger range, the profit of the supplier in the agency scenario increases less than the profit of the supplier in the basic scenario for every unit increase in the cost sensitivity to effort. In other words, cost sensitivity to effort also has less impact on the supplier’s profit in the agency scenario. Consequently, Corollary 2 reveals that when the cost sensitivity to effort is in a smaller range, the supplier is more profitable in the agency scenario. In contrast, when the cost sensitivity to effort is in a larger range, the basic scenario is more beneficial for the supplier.

#### 5.2.2 The property analysis for the optimal $\Pi_{ER_{1}}^{A}$. Property 11

The influence of *α* on the optimal profit of *ER*_1_ depends on the value range of *α*. Specifically, four cases are possible:

If $0<\xi_{1}<\frac{\beta }{2-\beta^{2}}$, $0<\xi_{2}<\frac{\xi_{1}(2-\beta^{2})}{\beta }$:

(i) When $0<\alpha <-\frac{B_{20}}{A_{20}}$, therefore, $\frac{\partial \Pi_{ER_{1}}^{A*}}{\partial \alpha }<0$; (ii) When $-\frac{B_{20}}{A_{20}}\leq \alpha <1$, therefore, $\frac{\partial \Pi_{ER_{1}}^{A*}}{\partial \alpha }\geq 0$.

If $0<\xi_{1}<\frac{\beta }{2-\beta^{2}}$, $\frac{\xi_{1}(2-\beta^{2})}{\beta }\leq \xi_{2}<1$:

(iii) When $0<\alpha <-\frac{B_{21}}{A_{21}}$, therefore, $\frac{\partial \Pi_{ER_{1}}^{A*}}{\partial \alpha }<0$; (iv) When $-\frac{B_{21}}{A_{21}}\leq \alpha <1$, therefore, $\frac{\partial \Pi_{ER_{1}}^{A*}}{\partial \alpha }\geq 0$. Property 11 implies that under the agency scenario, when the cost sensitivity to effort is in a smaller range, the optimal profit level of the new e-tailer will decrease with the cost sensitivity to effort. In contrast, if the cost sensitivity to effort is in a larger range, the new e-tailer’s optimal profit is positively correlated with the cost sensitivity to effort.

**Corollary 3. $\frac{\partial \Pi_{ER_{1}}^{A*}}{\partial \alpha }>\frac{\partial \Pi_{ER_{1}}^{B*}}{\partial \alpha }$**.

Similar to Corollary 2, by comparing the agency scenario with the basic scenario, we find that when the cost sensitivity to effort is in a smaller range, the profit of the new e-tailer in the agency scenario decreases less than the profit of the new e-tailer in the basic scenario for every unit increase in the cost sensitivity to effort. Namely, cost sensitivity to effort has less impact on the new e-tailer’s profit in the agency scenario. Conversely, when the cost sensitivity to effort is in a larger range, the profit of the new e-tailer in the agency scenario increases more than the profit of the new e-tailer in the basic scenario for every unit increase in the cost sensitivity to effort. In other words, cost sensitivity to effort has a greater impact on the new e-tailer’s profit in the agency scenario. As such, Corollary 3 reveals that regardless of the scope of the cost sensitivity to effort, the new e-tailer is always more profitable in the agency scenario.

#### 5.2.3 The property analysis for the optimal $\Pi_{ER_{2}}^{A}$. Property 12

Similar to property 11, the influence of *α* on the optimal profit of *ER*_2_ depends on the value range of *α*. Specifically, four cases are possible:

If $0<\xi_{2}<\frac{\beta }{2-\beta^{2}}$, $0<\xi_{1}<\frac{\xi_{2}(2-\beta^{2})}{\beta }$:

(i) When $0<\alpha <-\frac{B_{22}}{A_{22}}$, therefore, $\frac{\partial \Pi_{ER_{2}}^{A*}}{\partial \alpha }<0$; (ii) When $-\frac{B_{22}}{A_{22}}\leq \alpha <1$, therefore, $\frac{\partial \Pi_{ER_{2}}^{A*}}{\partial \alpha }\geq 0$.

If $0<\xi_{2}<\frac{\beta }{2-\beta^{2}}$, $\frac{\xi_{2}(2-\beta^{2})}{\beta }\leq \xi_{1}<1$:

(iii) When $0<\alpha <-\frac{B_{23}}{A_{23}}$, therefore, $\frac{\partial \Pi_{ER_{2}}^{A*}}{\partial \alpha }<0$; (iv) When $-\frac{B_{23}}{A_{23}}\leq \alpha <1$, therefore, $\frac{\partial \Pi_{ER_{2}}^{A*}}{\partial \alpha }\geq 0$. Property 12 implies that under the agency scenario, when the cost sensitivity to effort is in a smaller range, the optimal profit level of the traditional e-tailer will decrease with the cost sensitivity to effort. In contrast, if the cost sensitivity to effort is in a larger range, the traditional e-tailer’s optimal profit is positively correlated with the cost sensitivity to effort.

**Corollary 4. $\frac{\partial \Pi_{ER_{2}}^{A*}}{\partial \alpha }>\frac{\partial \Pi_{ER_{2}}^{E*}}{\partial \alpha }$**.

Similar to Corollary 3, by comparing the agency scenario with the basic scenario, we find that when the cost sensitivity to effort is in a smaller range, the profit of the traditional e-tailer in the agency scenario decreases less than the profit of the traditional e-tailer in the basic scenario for every unit increase in the cost sensitivity to effort. Namely, cost sensitivity to effort has less impact on the traditional e-tailer’s profit in the agency scenario. Conversely, when the cost sensitivity to effort is in a larger range, the profit of the traditional e-tailer in the agency scenario increases more than the profit of the traditional e-tailer in the basic scenario for every unit increase in the cost sensitivity to effort. In other words, cost sensitivity to effort has a greater impact on the traditional e-tailer’s profit in the agency scenario. As such, Corollary 4 reveals that regardless of the scope of the cost sensitivity to effort, the traditional e-tailer is always more profitable in the agency scenario.

As we can see from properties and corollaries in the extended model, the agency scenario and the basic scenario are very similar. Consequently, the other managerial findings and insights that arise under the basic model will continue to hold under the agency model. In addition, the most important we find is that the e-tailers are always more profitable in the agency model regardless of the scope of the cost sensitivity to effort. Namely, compared to the basic model, the agency model may be a more efficient way to improve the benefits of e-tailers. However, as fa as the supplier is concerned, the agency scenario is not always favourable. Specifically, when the cost sensitivity to effort is in a smaller range, the supplier is more profitable in the agency scenario. In contrast, when the cost sensitivity to effort is in a larger range, the basic scenario is more beneficial for the supplier.

## 6. Numerical examples and analysis

To show the property and comparison results more clearly, we provide some numerical examples to confirm the results derived in the previous sections and display the performance of the proposed blockchain technology by comparison with two e-tail channel models. To ensure that the parameters are set appropriately and all the conditions assumed in the previous section are satisfied, we refer to the empirical estimations reported in the literature (e.g., Cole et al. [12], Sunny et al. [19], van Hoek [58]) as well as the parameter estimations from the literature of game theory (e.g., Liu et al. [24], Liu et al. [25], Genc and De Giovanni [59]). Specifically, Genc and De Giovanni [59] perform an numerical examples of the game results by setting the relevant parameters, clearly presenting the propositional results and verifying the accuracy of the conclusions. As such, we suppose that the values of the parameters are as follows: *a* = 100, *p* = 5. As stated in Section 3, the settings of these parameters need to satisfy the following conditions: (i) 0<*θ*<1; (ii) 0<*α*<1; (iii) 0<*β*<1; and (iv) *γ*_*b*_≥0.

### 6.1 Numerical analysis of the decision variables

This section mainly examines the property in Section 5. To further clearly present how parameters (*α*, *β*, *θ*) affect decision variables (i.e., w*, $t_{b}^{*}$ and $t_{e}^{*}$) and the profit level of the supplier and the two e-tailers (i.e., $\Pi_{S}^{*}$, $\Pi_{ER_{1}}^{B*}$ and $\Pi_{ER_{2}}^{E*}$), we will perform a specific numerical analysis for the properties of decision variables and profits.

#### 6.1.1 Numerical analysis of the optimal *W*

**Fig 2:**
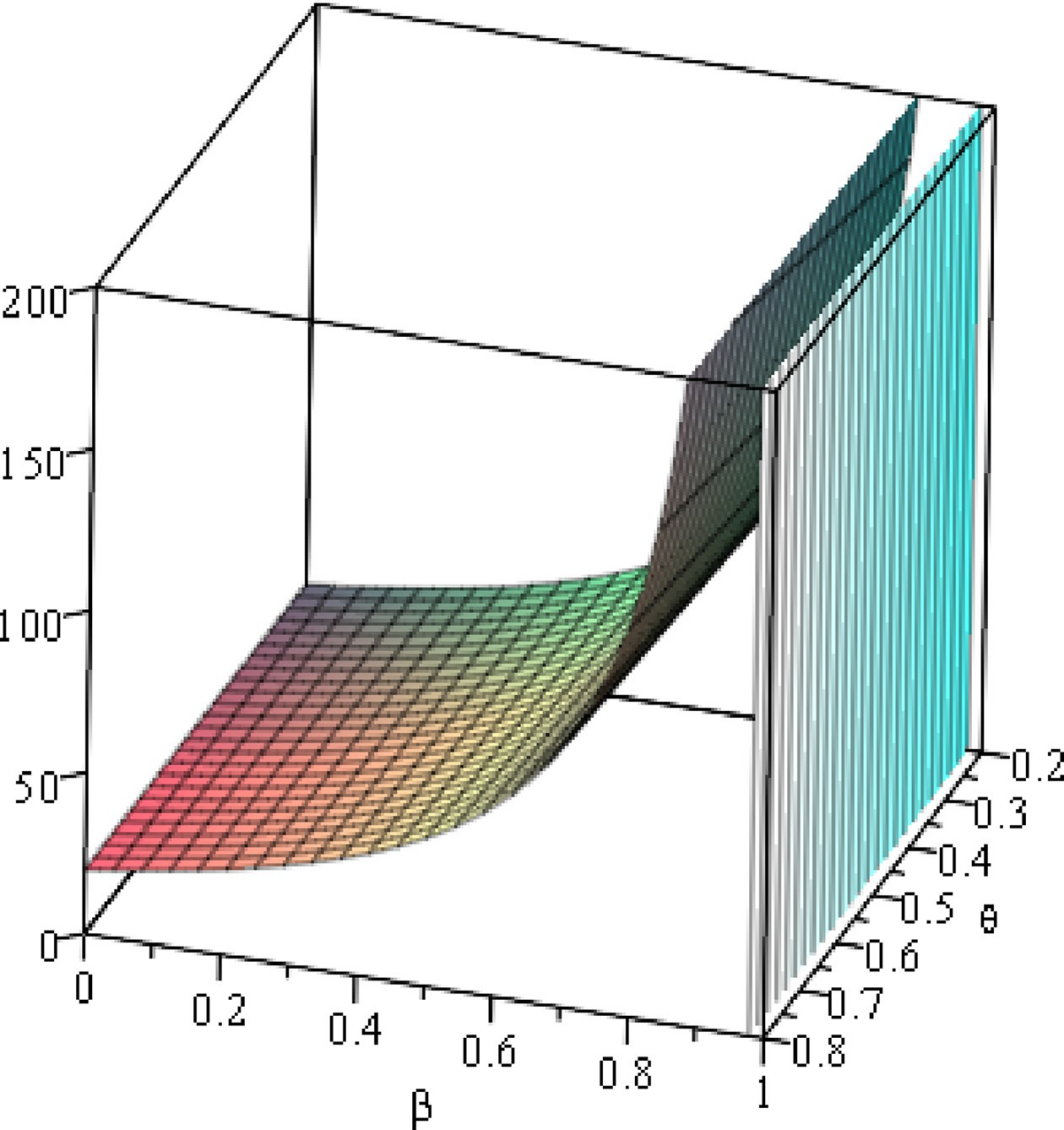
Relationship between $\frac{\partial w^{*}}{\partial \alpha }$ and *β*/*θ*.

**Fig 3:**
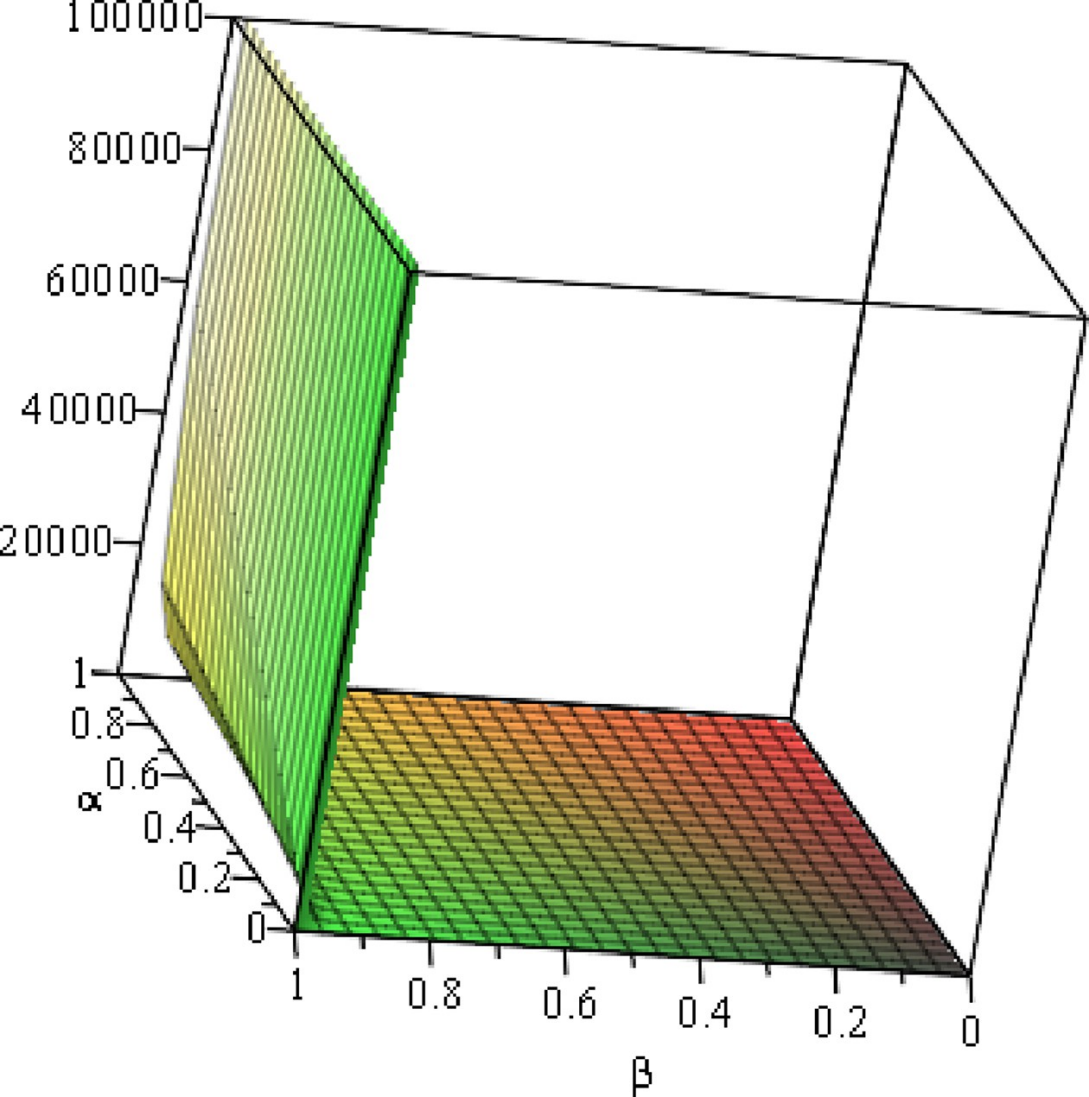
Relationship between $\frac{\partial w^{*}}{\partial \beta }$ and *α*/*β*. (*a* = 100) (*a* = 100, *p* = 5, *θ* = 0.4).

**Fig 4:**
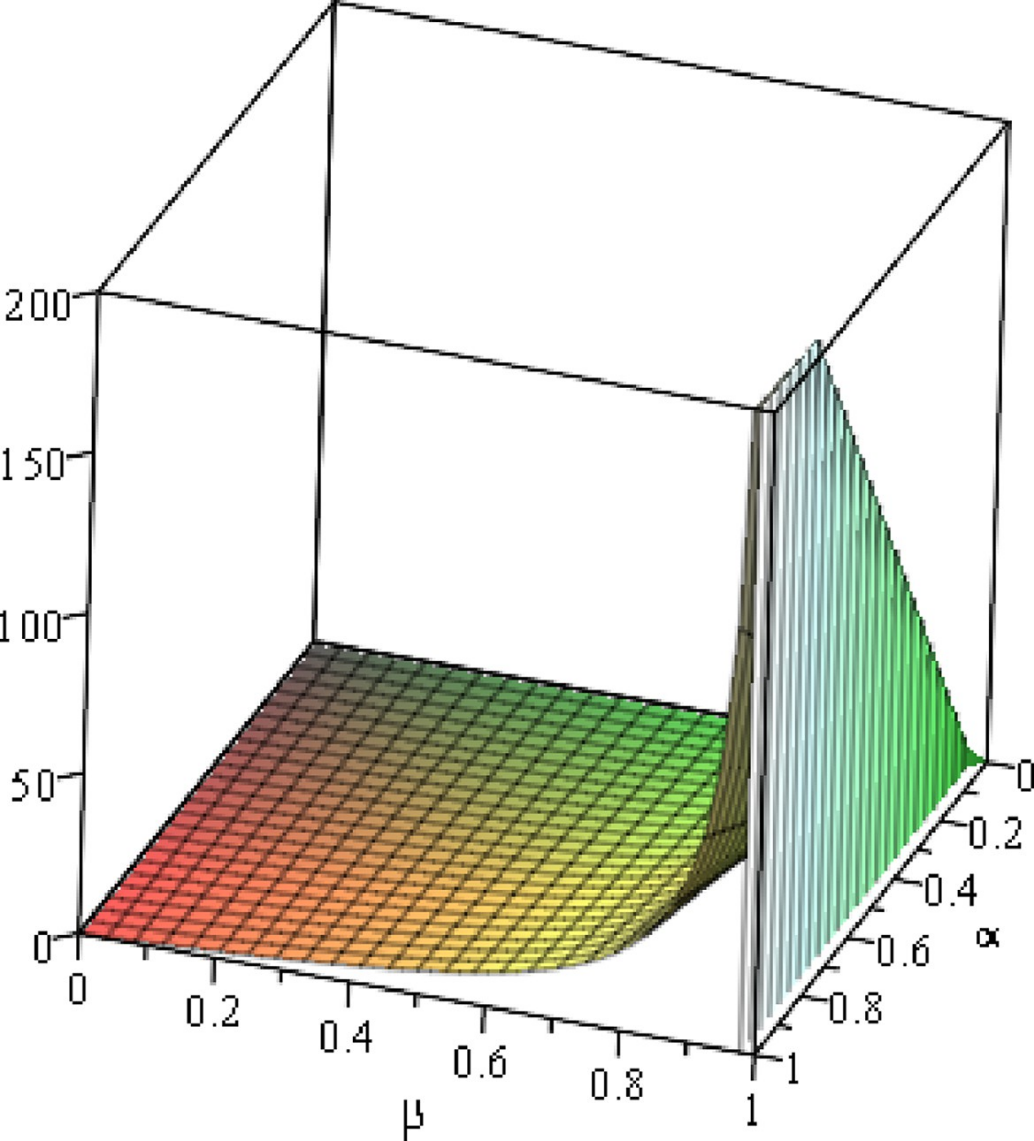
Relationship between $\frac{\partial w^{*}}{\partial \theta }$ and *α*/*β*. (*a* = 100).

To clearly present the impact of the cost sensitivity to effort (*α*), cross-effort elasticity coefficient of demand (*β*) and the market share of blockchain-sensitive consumer (*θ*) on the optimal wholesale price (*w*), the relationship between $\frac{\partial w^{*}}{\partial \alpha }$ and *β***/***θ*, relationship between $\frac{\partial w^{*}}{\partial \alpha }$ and *β***/***θ*, and relationship between $\frac{\partial w^{*}}{\partial \theta }$ and *α***/***β* will be presented by fixing *a* = 100, *p* = 5. As shown in Figs 2–4.

The conclusion in Property 1 is demonstrated in Fig 2, showing the relationship between $\frac{\partial w^{*}}{\partial \alpha }$ and *β*/*θ*, and we see that the result is always positive. Specifically, setting *a* = 100, regardless of how the cross-effort elasticity coefficient of demand *β* (0<*β*<1) or the market share of blockchain-sensitive consumer *θ* (0<*θ*<1) changes, the improvement of the cost sensitivity to effort *α* (0<*α*<1) always increases the optimal wholesale price. Fig 3 shows the relationship between $\frac{\partial w^{*}}{\partial \beta }$ and *α*/*β*, and we can also easily find that the result is always positive. Specifically, setting *a* = 100, *p* = 5, *θ* =0.4, regardless of how the cost sensitivity to effort *α* or the cross-effort elasticity coefficient of demand *β* changes, the optimal wholesale price always increases with the improvement of the cross-effort elasticity coefficient of demand *β*. Similarly, Fig 4 shows that the optimal wholesale price grows as the market share of blockchain-sensitive consumer *θ* increases, regardless of whether the cost sensitivity to effort *α* or cross-effort elasticity coefficient of demand *β* changes.

#### 6.1.2 Numerical analysis of the optimal *t*_*b*_

**Fig 5:**
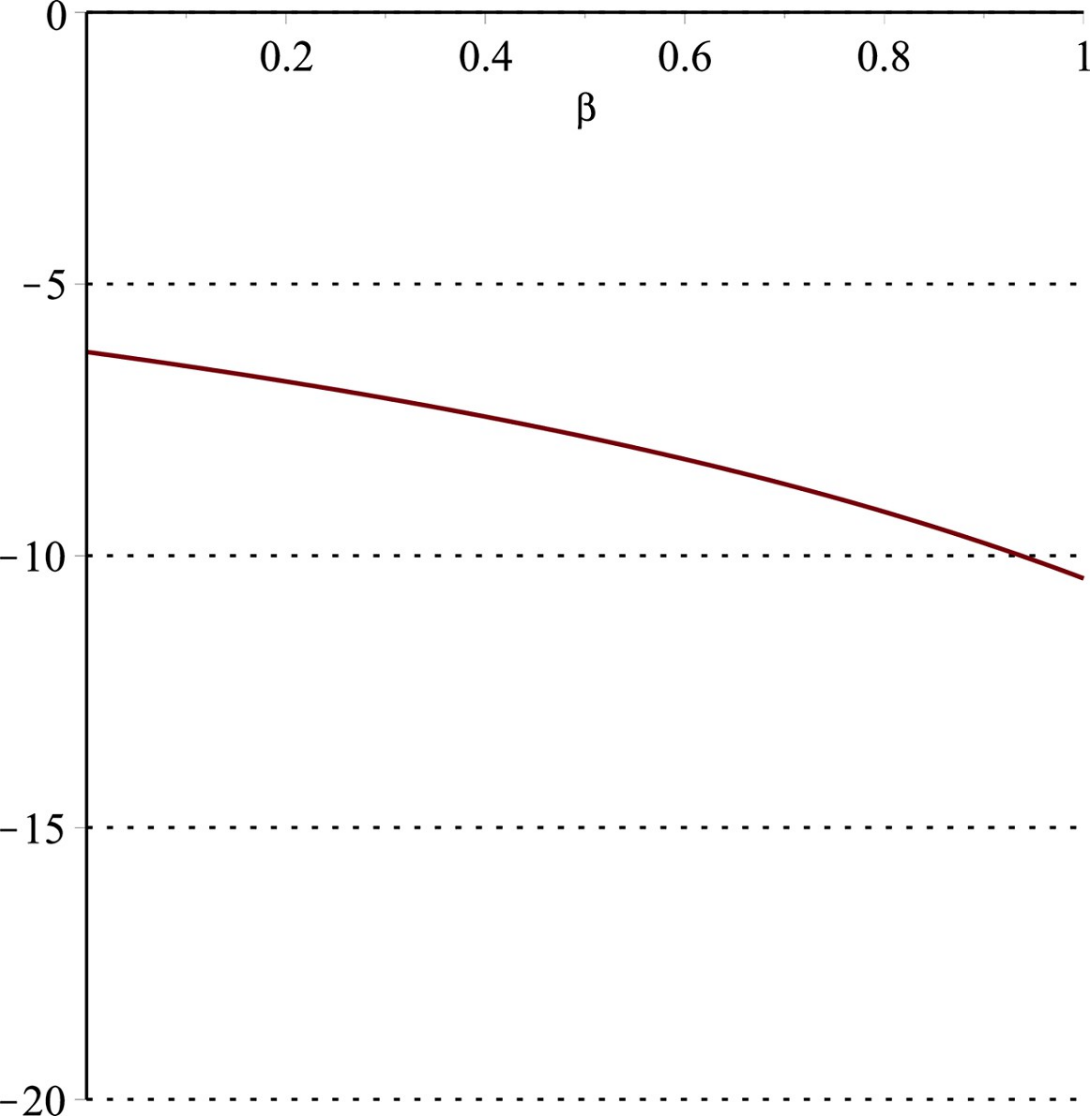
Relationship between $\frac{\partial t_{b}^{*}}{\partial \alpha }$ and *β*.

**Fig 6:**
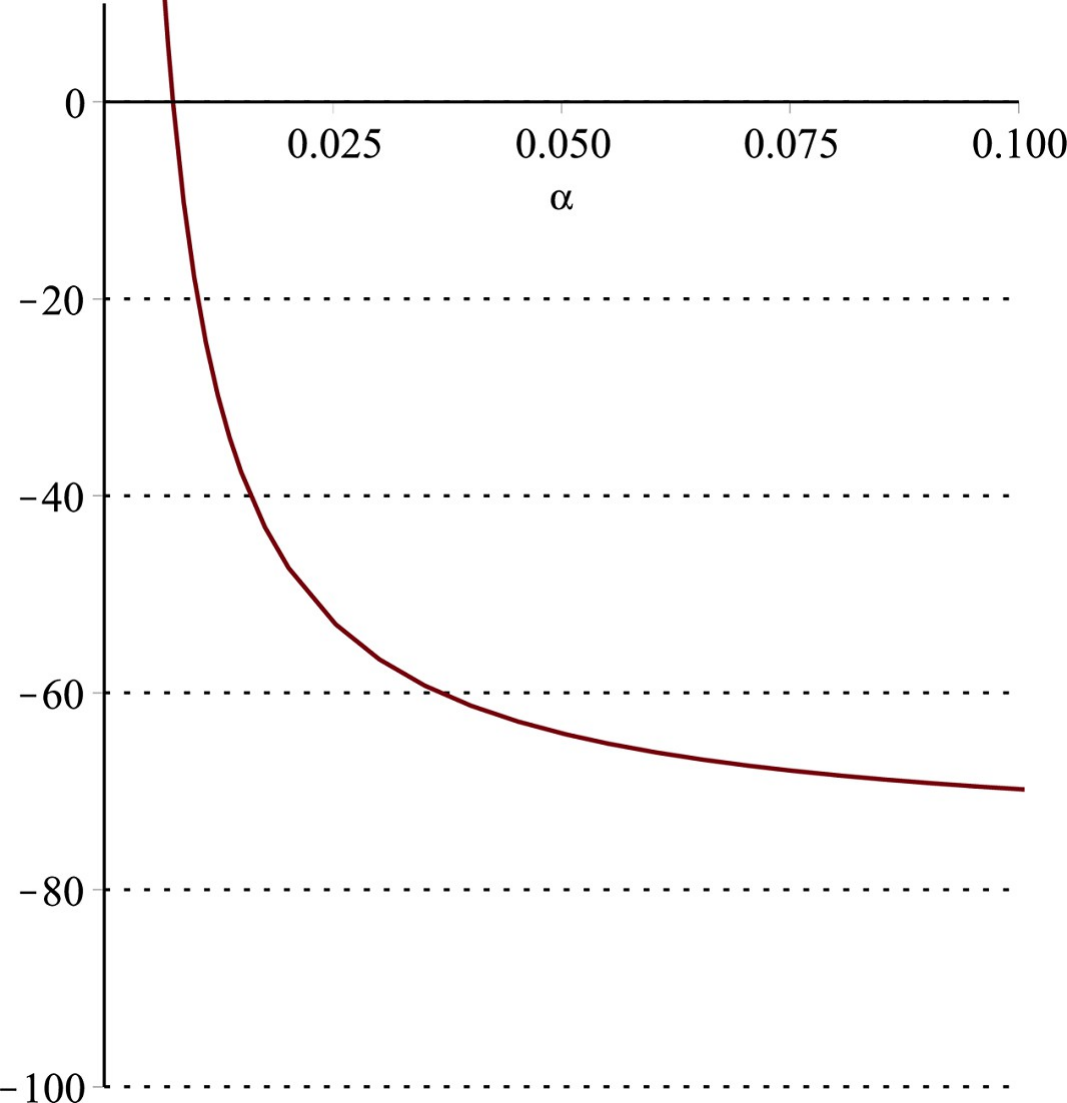
Relationship between $\frac{\partial t_{b}^{*}}{\partial \beta }$ and *α*. (*a* = 100, *p* = 5, *α* = 0.4) (*a* = 100, *p* = 5, *β* = 0.4, *θ* = 0.4).

**Fig 7:**
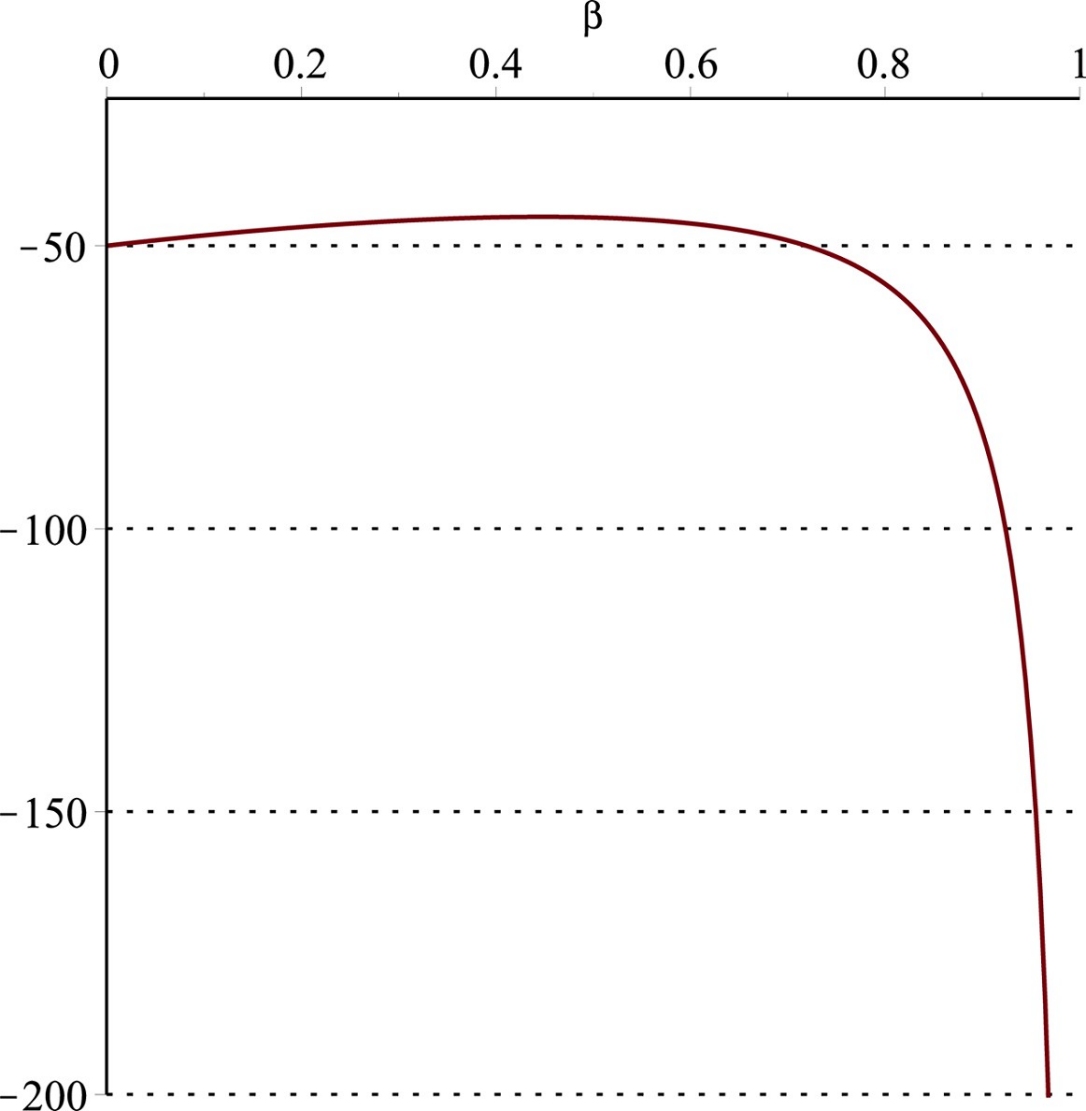
Relationship between $\frac{\partial t_{b}^{*}}{\partial \theta }$ and *β*. (*a* = 100).

To clearly present the impact of the cost sensitivity to effort (*α*), cross-effort elasticity coefficient of demand (*β*) and the market share of blockchain-sensitive consumer (*θ*) on the optimal effort with blockchain of *ER*_1_ (*t*_*b*_), the relationship between $\frac{\partial t_{b}^{*}}{\partial \alpha }$ and *β*, relationship between $\frac{\partial t_{b}^{*}}{\partial \beta }$ and *α*, and relationship between $\frac{\partial t_{b}^{*}}{\partial \theta }$ and *β* will be presented by fixing *a* = 100, *p* = 5. As shown in Figs 5–7.

The conclusion in Property 2 is demonstrated in Fig 5, showing the relationship between $\frac{\partial t_{b}^{*}}{\partial \alpha }$ and *β*; we see that the result is always negative. Specifically, setting *a* = 100, *p* = 5, *α* =0.4, no matter how the cross-effort elasticity coefficient of demand *β* (0<*β*<1) changes, the improvement of the cost sensitivity to effort *α* (0<*α*<1) does not increase the effort with a blockchain of *ER*_1_. Fig 6 shows the relationship between $\frac{\partial t_{b}^{*}}{\partial \beta }$ and *α*. Specifically, setting *a* = 100, *p* = 5, *β* = 0.4, *θ* = 0.4,, when the cost sensitivity to the effort *α* is lower than a threshold (*α* = 0.0075), the improvement of the cross-effort elasticity coefficient of demand *β* increases the effort with a blockchain of *ER*_1_. Once the cost sensitivity to effort *α* exceeds the threshold, the effort with blockchain of *ER*_1_ will not grow as cross-effort elasticity coefficient of demand *β* increases. Fig 7 shows that the effort with blockchain of *ER*_1_ decreases as the market share of blockchain-sensitive consumer *θ* (0<*θ*<1) increases, regardless of whether the cross-effort elasticity coefficient of demand *β* changes.

#### 6.1.3 Numerical analysis of the optimal *t*_*e*_

**Fig 8:**
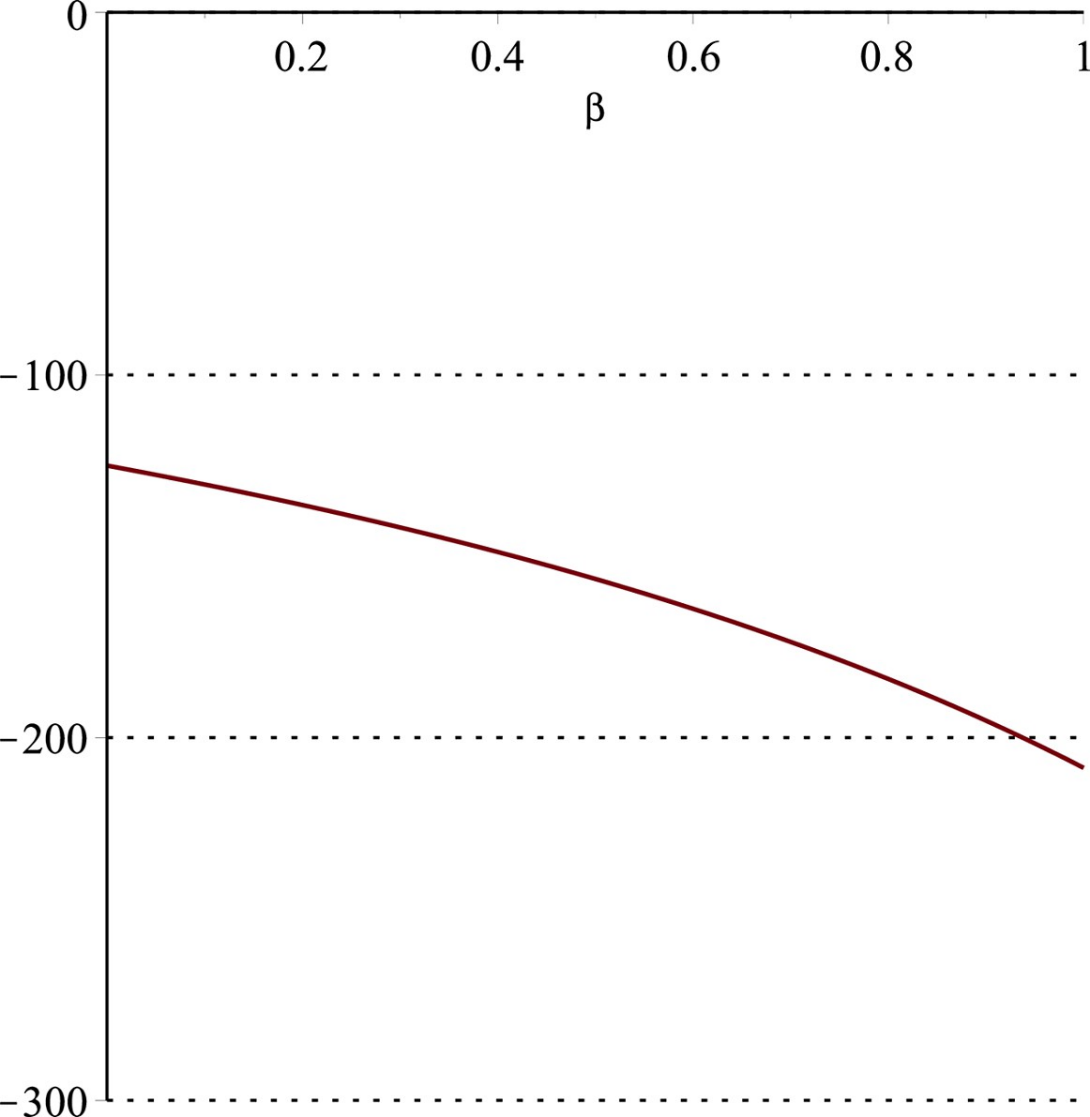
Relationship between $\frac{\partial t_{e}^{*}}{\partial \alpha }$ and *β*.

**Fig 9:**
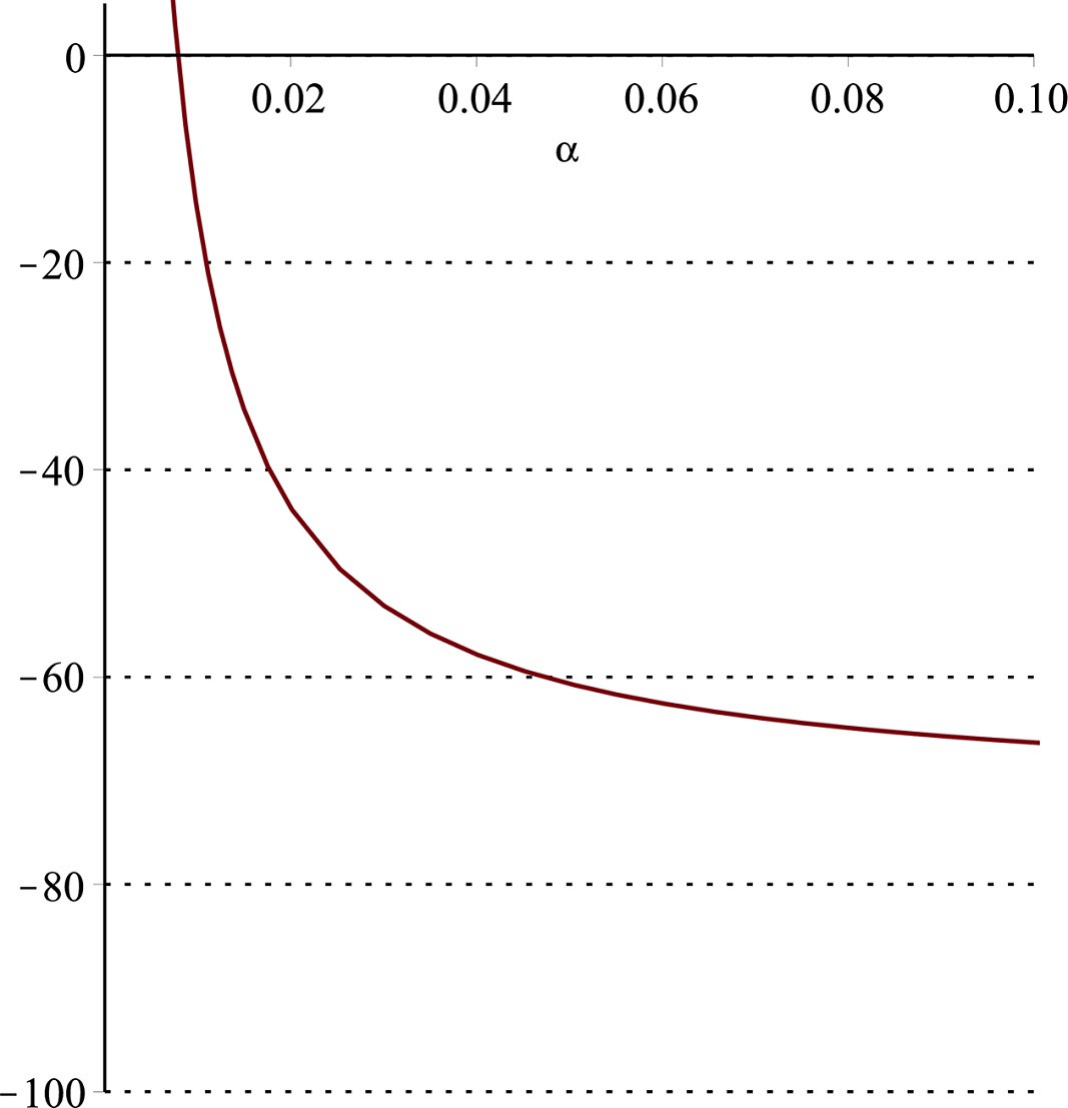
Relationship between $\frac{\partial t_{e}^{*}}{\partial \beta }$ and *α*. (*a* = 100, *p* = 5, *α* = 0.4) (*a* = 100, *p* = 5, *β* = 0.4, *θ* = 0.4).

**Fig 10:**
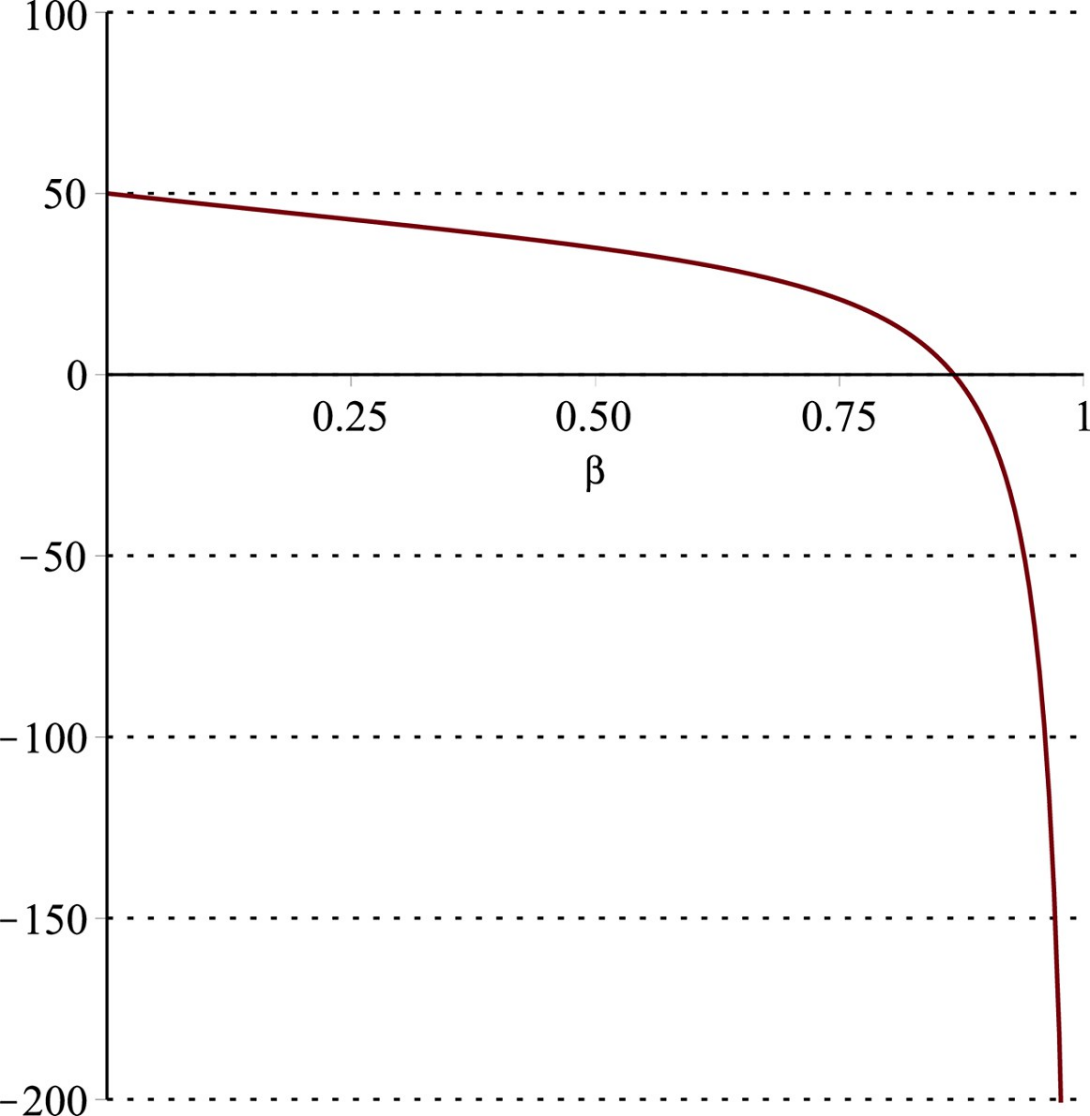
Relationship between $\frac{\partial t_{e}^{*}}{\partial \theta }$ and *β*. (*a* = 100).

To clearly present the impact of the cost sensitivity to effort (*α*), cross-effort elasticity coefficient of demand (*β*) and the market share of blockchain-sensitive consumer (*θ*) on the optimal effort without blockchain of *ER*_2_ (*t*_*e*_), the relationship between $\frac{\partial t_{e}^{*}}{\partial \alpha }$ and *β*, relationship between $\frac{\partial t_{e}^{*}}{\partial \beta }$ and *α*, and relationship between $\frac{\partial t_{e}^{*}}{\partial \theta }$ and *β* will be presented by fixing *a* = 100, *p* = 5. As shown in Figs 8–10.

We can easily see that $\frac{\partial t_{e}^{*}}{\partial \alpha }<0$ in Fig 8. Specifically, in Fig 8, setting *a* = 100, *p* = 5, *α* = 0.4, no matter how the cross-effort elasticity coefficient of demand *β* (0<*β*<1) changes, effort without blockchain of *ER*_2_ always decreases when the cost sensitivity to the effort *α* (0<*α*<1) increases. In Fig 9, setting *a* = 100, *p* = 5, *β* = 0.4, *θ* = 0.4, when *α* (0<*α*<0.0079) is low enough, effort without blockchain of *ER*_2_ increases with the increases of the cost sensitivity to the effort *β*; however, when *α* (0.0079≤*α*<1) is not low enough, the improvement of the cross-effort elasticity coefficient of demand *β* (0<*β*<1) does not increase the effort without blockchain of *ER*_2_. In Fig 10, setting *a* = 100, when *β* (0.8672≤*β*<1) is high enough, the improvement of the market share of blockchain-sensitive consumer *θ* (0<*θ*<1) always decreases the effort without blockchain of *ER*_2_; in contrast, when *β* (0<*β*<0.8672) is not high enough, the improvement of the market share of blockchain-sensitive consumer *θ* (0<*θ*<1) always increases the effort without blockchain of *ER*_2_.

### 6.2 Numerical analysis of the optimal profits

#### 6.2.1 Numerical analysis of the optimal Π_*S*_

**Fig 11:**
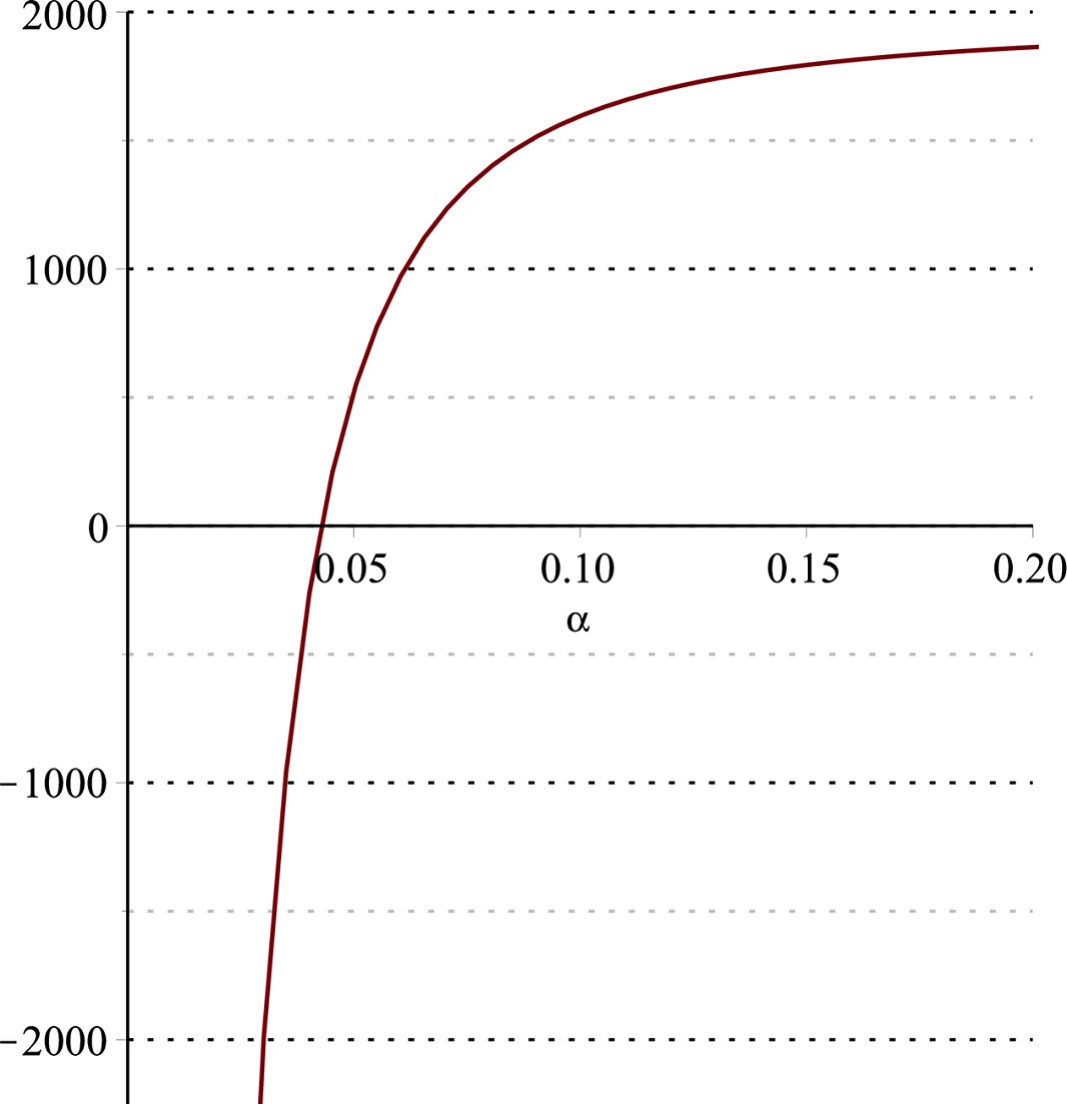
Relationship between $\frac{\partial \Pi_{S}^{*}}{\partial \alpha }$ and *α*.

**Fig 12:**
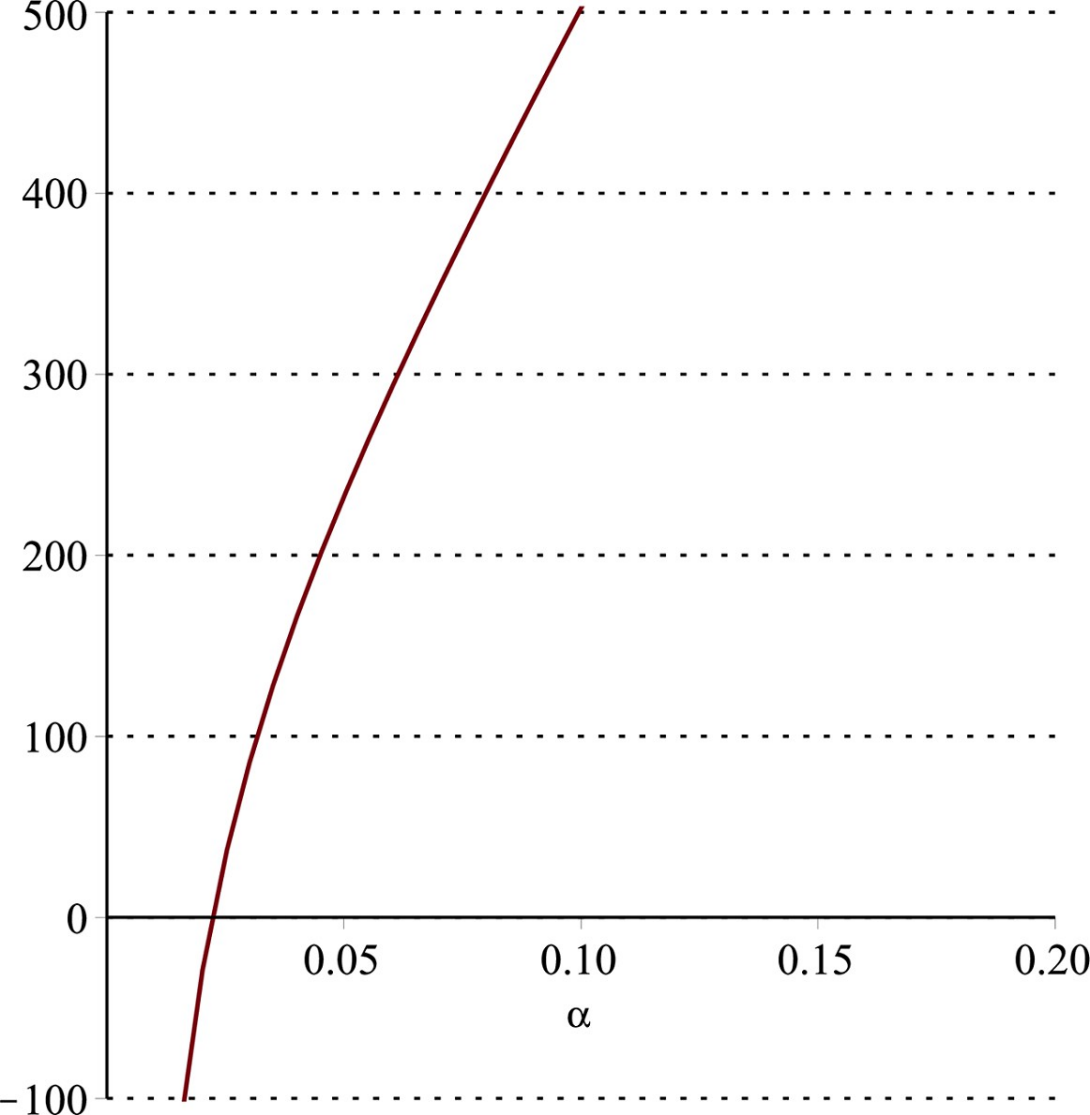
Relationship between $\frac{\partial \Pi_{S}^{*}}{\partial \beta }$ and *α*. (*a* = 100, *p* = 5, *β* = 0.4, *θ* = 0.4) (*a* = 100, *p* = 5, *β* = 0.4, *θ* = 0.4).

**Fig 13:**
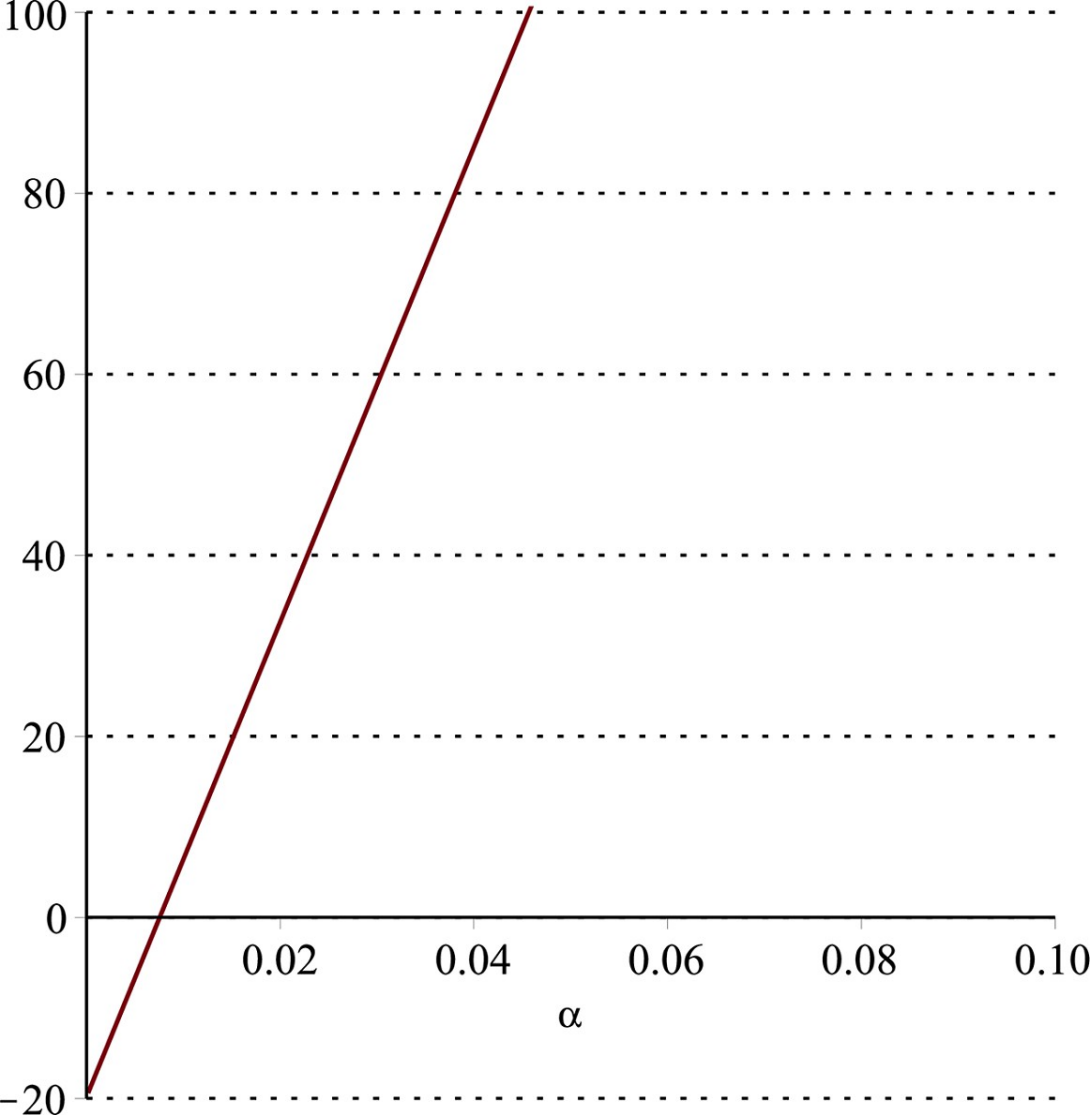
Relationship between $\frac{\partial \Pi_{S}^{*}}{\partial \theta }$ and α. (a = 100, p = 5, β = 0.4, θ = 0.4).

To clearly present the impact of the cost sensitivity to effort (*α*), cross-effort elasticity coefficient of demand (*β*) and the market share of blockchain-sensitive consumer (*θ*) on the optimal profits of supplier (Π_*S*_), the relationship between $\frac{\partial \Pi_{S}^{*}}{\partial \alpha }$ and *α*, relationship between $\frac{\partial \Pi_{S}^{*}}{\partial \beta }$ and *α*, and relationship between $\frac{\partial \Pi_{S}^{*}}{\partial \theta }$ and *α* will be presented by fixing *a* = 100, *p* = 5, *β* = 0.4, *θ* = 0.4. As shown in Figs 11–13.

Setting *a* = 100, *p* = 5, *β* = 0.4, *θ* = 0.4 by comparing the three pictures, it is easy to find that when *α* is low enough, $\frac{\partial \Pi_{S}^{*}}{\partial \alpha }<0$, $\frac{\partial \Pi_{S}^{*}}{\partial \beta }<0$, $\frac{\partial \Pi_{S}^{*}}{\partial \theta }<0$; when *α* is not low enough, $\frac{\partial \Pi_{S}^{*}}{\partial \alpha }\geq 0$, $\frac{\partial \Pi_{S}^{*}}{\partial \beta }\geq 0$, $\frac{\partial \Pi_{S}^{*}}{\partial \theta }\geq 0$. Specifically, in Fig 11, when *α* (0<*α*<0.0428) is low enough, the supplier’s profits decrease with increasing cost sensitivity to effort *α*; when *α* (0.0428≤*α*<1) is not low enough, the supplier’s profits increase with increasing cost sensitivity to effort *α*. In Fig 12, when the cost sensitivity to effort *α* is lower than a threshold (*α* = 0.0222), the improvement of the cross-effort elasticity coefficient of demand *β* (0<*β*<1) decreases the supplier’s profits. Once the cost sensitivity to effort *α* exceeds the threshold, the supplier’s profits will grow as the cross-effort elasticity coefficient of demand *β* increases. Similarly, in Fig 13, when the cost sensitivity to effort *α* is lower than a threshold (*α* = 0.0076), the improvement of the market share of blockchain-sensitive consumer *θ* (0<*θ*<1) decreases the supplier’s profits. Once the cost sensitivity to effort *α* exceeds the threshold, the supplier’s profits will grow as the market share of blockchain-sensitive consumer *θ* increases.

#### 6.2.2 Numerical analysis of the optimal $\Pi_{ER_{1}}^{B}$

**Fig 14:**
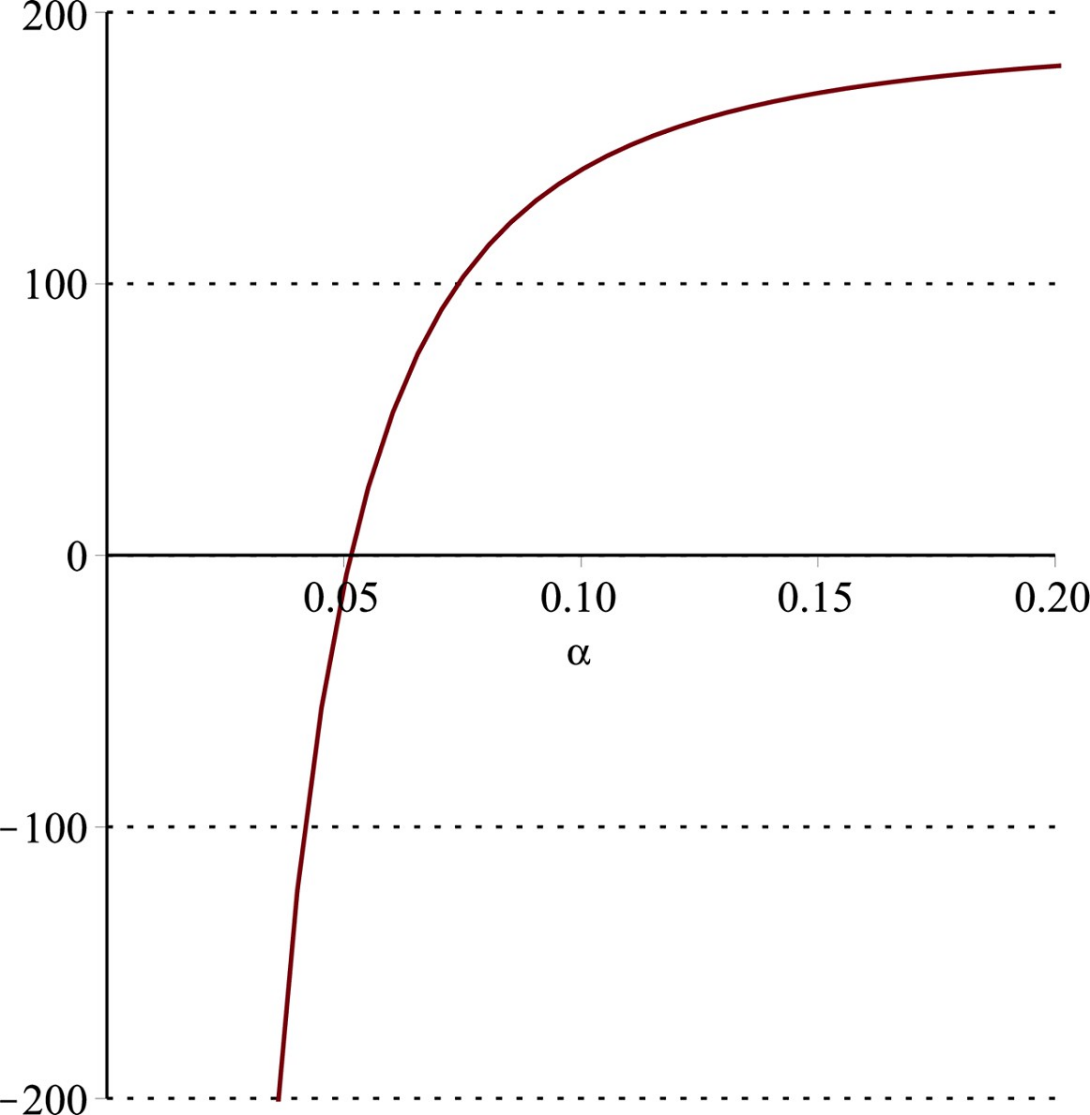
Relationship between $\frac{\partial \Pi_{ER_{1}}^{B*}}{\partial \alpha }$ and *α*.

**Fig 15:**
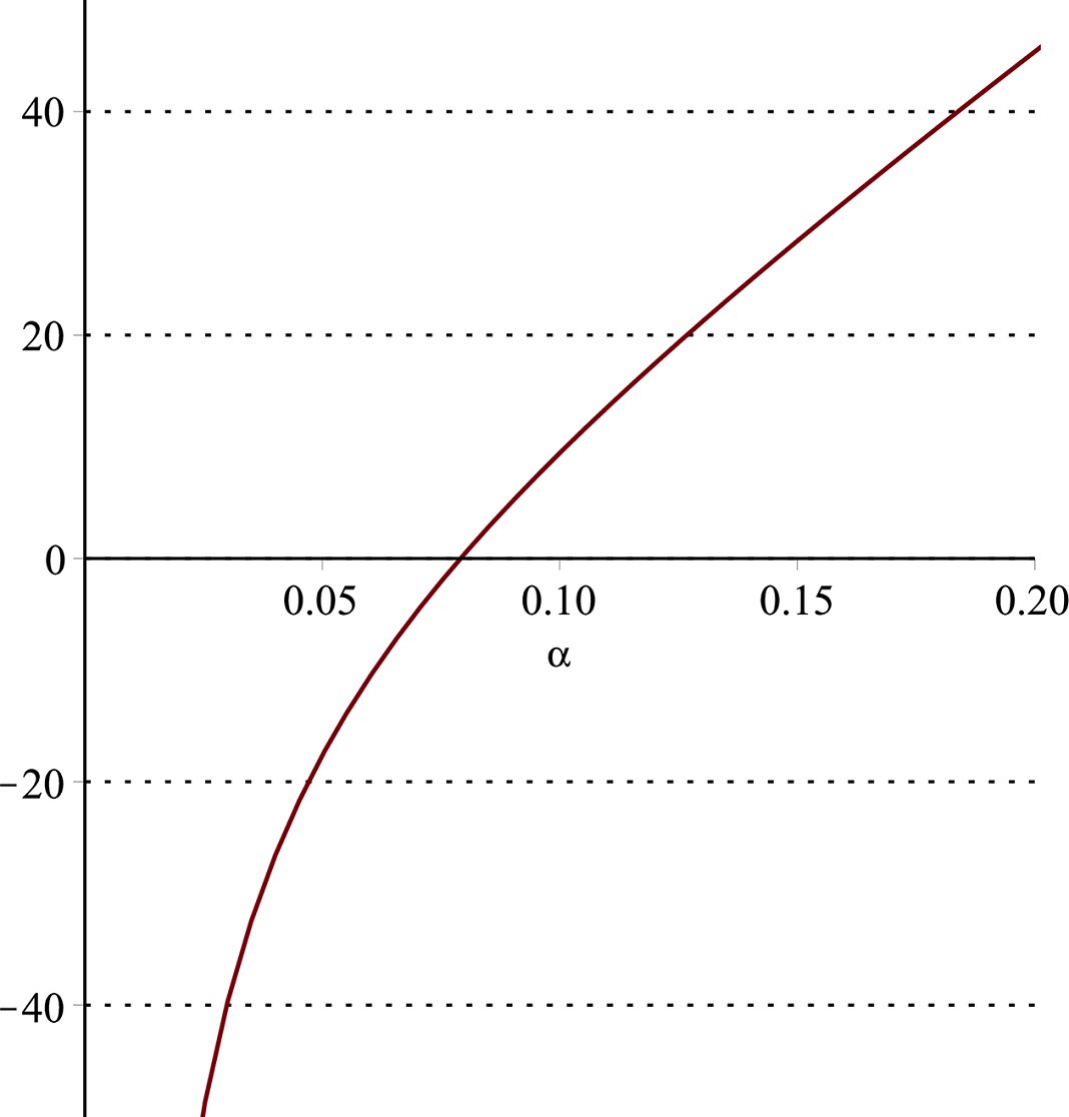
Relationship between $\frac{\partial \Pi_{ER_{1}}^{B*}}{\partial \beta }$ and *α*. (*a* = 100, *p* = 5, *β* = 0.4, *θ* = 0.4) (*a* = 100, *p* = 5, *β* = 0.4, *θ* = 0.4).

**Fig 16:**
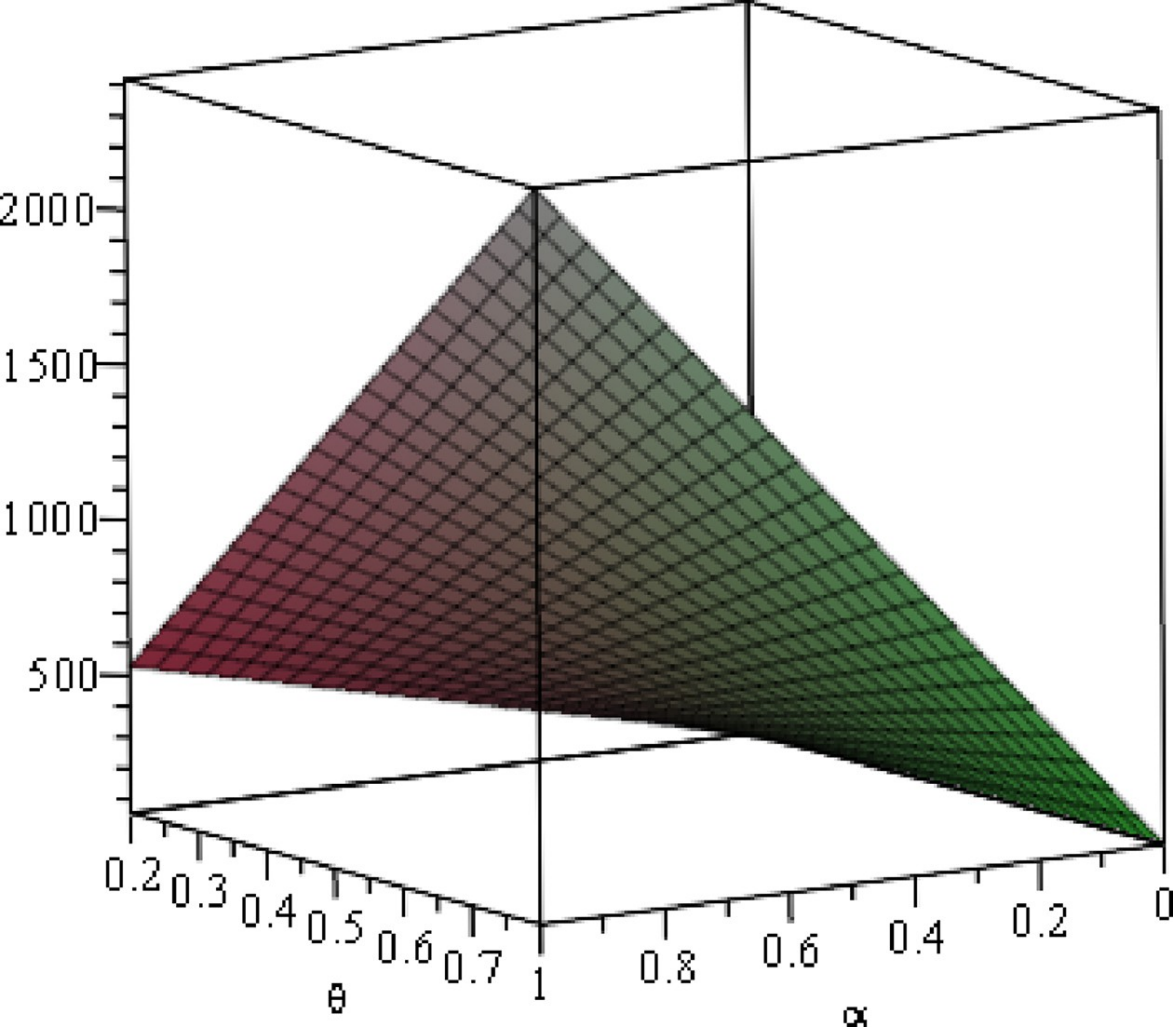
Relationship between $\frac{\partial \Pi_{ER_{1}}^{B*}}{\partial \theta }$ and *α*/*θ*. (*a* = 100, *p* = 5, *β* = 0.4).

To clearly present the impact of the cost sensitivity to effort (*α*), cross-effort elasticity coefficient of demand (*β*) and the market share of blockchain-sensitive consumer (*θ*) on the optimal profits of *ER*_1_ ($\Pi_{ER_{1}}^{B}$), the relationship between $\frac{\partial \Pi_{ER_{1}}^{B*}}{\partial \alpha }$ and *α*, relationship between $\frac{\partial \Pi_{ER_{1}}^{B*}}{\partial \beta }$ and *α*, and relationship between $\frac{\partial \Pi_{ER_{1}}^{B*}}{\partial \theta }$ and *α***/***θ* will be presented by fixing *a* = 100, *p* = 5, *β* = 0.4. As shown in Figs 14–16.

Setting *a* = 100, *p* = 5, *β* = 0.4, *θ* = 0.4, by comparing Figs 14 and 15, it is easy to find that when *α* is low enough, $\frac{\partial \Pi_{ER_{1}}^{B*}}{\partial \alpha }<0$, $\frac{\partial \Pi_{ER_{1}}^{B*}}{\partial \beta }<0$; when *α* is not low enough, $\frac{\partial \Pi_{ER_{1}}^{B*}}{\partial \alpha }\geq 0$, $\frac{\partial \Pi_{ER_{1}}^{B*}}{\partial \beta }\geq 0$. Specifically, in Fig 14, when the cost sensitivity to effort *α* is lower than a threshold (*α* = 0.0514), the improvement of the cost sensitivity to effort *α* decreases the profits of retailer *ER*_1_. Once the cost sensitivity to effort *α* exceeds the threshold, the profits of retailer *ER*_1_ will grow as the cost sensitivity to effort *α* increases. Similarly, in Fig 15, when the cost sensitivity to effort *α* is lower than a threshold (*α* = 0.0791), the improvement of the cross-effort elasticity coefficient of demand *β* (0<*β*<1) decreases the profits of retailer *ER*_1_. Once the cost sensitivity to effort *α* exceeds the threshold, the profits of retailer *ER*_1_ will grow as the cross-effort elasticity coefficient of demand *β* increases. In Fig 16, setting *a* = 100, p = 5, *β* = 0.4, we know that regardless of how *α* (0<*α*<1) or *θ* (0<*θ*<1)changes, the profits of retailer *ER*_1_ always grow as the market share of blockchain-sensitive consumer *θ* increases.

#### 6.2.3 Numerical analysis of the optimal $\Pi_{ER_{2}}^{E}$

**Fig 17:**
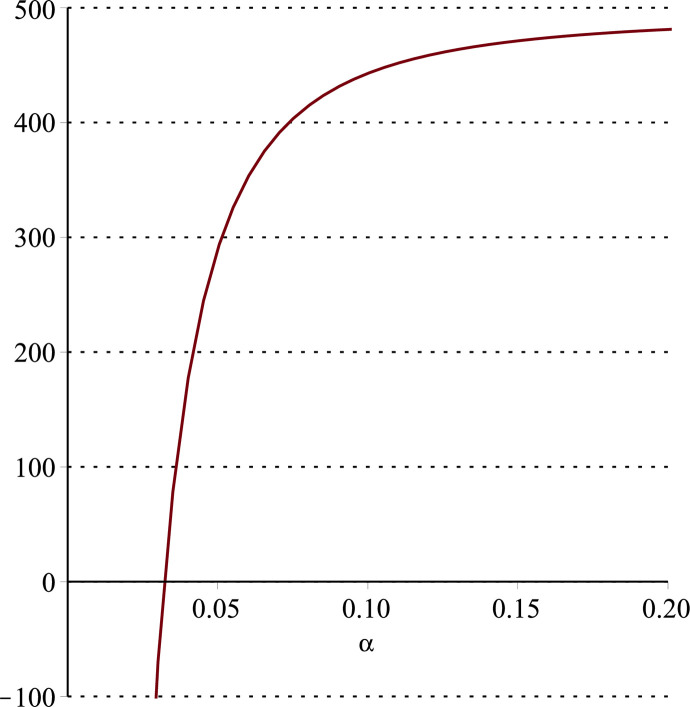
Relationship between $\frac{\partial \Pi_{ER_{2}}^{E*}}{\partial \alpha }$ and *α*.

**Fig 18:**
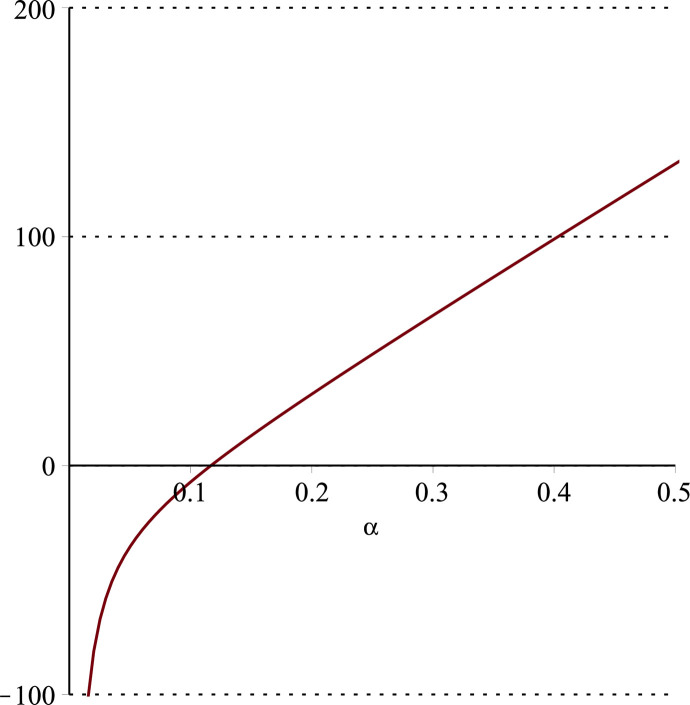
Relationship between $\frac{\partial \Pi_{ER_{2}}^{E*}}{\partial \beta }$ and *α*. (*a* = 100, *p* = 5, *β* = 0.4, *θ* = 0.4) (*a* = 100, *p* = 5, *β* = 0.4, *θ* = 0.4).

**Fig 19:**
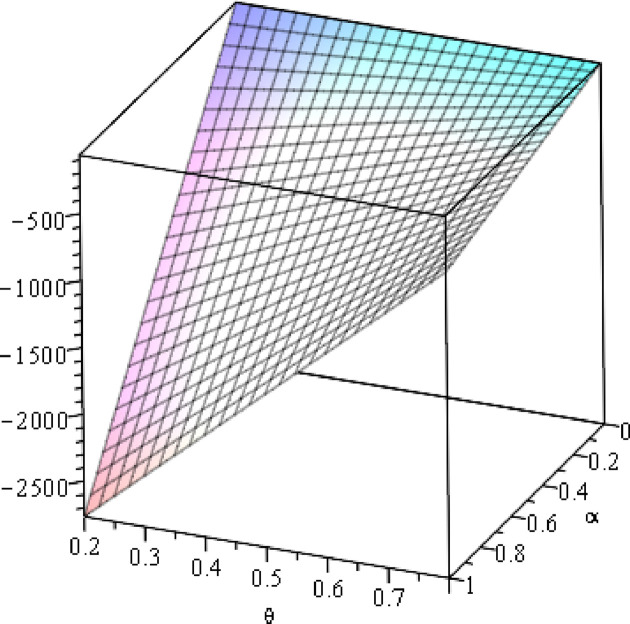
Relationship between $\frac{\partial \Pi_{ER_{2}}^{E*}}{\partial \theta }$ and *α*/*θ*. (*a* = 100, *p* = 5, *β* = 0.4).

To clearly present the impact of the cost sensitivity to effort (*α*), cross-effort elasticity coefficient of demand (*β*) and the market share of blockchain-sensitive consumer (*θ*) on the optimal profits of *ER*_2_ ($\Pi_{ER_{2}}^{E}$), the relationship between $\frac{\partial \Pi_{ER_{2}}^{E*}}{\partial \alpha }$ and *α*, relationship between $\frac{\partial \Pi_{ER_{2}}^{E*}}{\partial \beta }$ and *α*, and relationship between $\frac{\partial \Pi_{ER_{2}}^{E*}}{\partial \theta }$ and *α***/***θ* will be presented by fixing *a* = 100, *p* = 5, *β* = 0.4. As shown in Figs 17–19.

Setting *a* = 100, *p* = 5, *β* = 0.4, *θ* =0.4, by comparing Figs 17 and 18, it is easy to find that when *α* is low enough, $\frac{\partial \Pi_{ER_{2}}^{E*}}{\partial \alpha }<0$, $\frac{\partial \Pi_{ER_{2}}^{E*}}{\partial \beta }<0$; when *α* is not low enough, $\frac{\partial \Pi_{ER_{2}}^{E*}}{\partial \alpha }\geq 0$, $\frac{\partial \Pi_{ER_{2}}^{E*}}{\partial \beta }\geq 0$. Specifically, in Fig 17, when the cost sensitivity to effort *α* is lower than a threshold (*α* = 0.0321), the improvement of the cost sensitivity to effort *α* decreases the profits of retailer *ER*_2_. Once the cost sensitivity to effort *α* exceeds the threshold, the profits of retailer *ER*_2_ will grow as the cost sensitivity to effort *α* increases. Similarly, in Fig 18, when the cost sensitivity to effort *α* is lower than a threshold (*α* = 0.1169), the improvement of the cross-effort elasticity coefficient of demand *β* (0<*β*<1) decreases the profits of retailer *ER*_2_ Once the cost sensitivity to effort *α* exceeds the threshold, the profits of retailer *ER*_2_ will grow as the cross-effort elasticity coefficient of demand *β* increases. In Fig 19, setting *a* = 100, *p* = 5, *β* = 0.4, we know that regardless of how *α* (0<*α*<1) or *θ* (0<*θ*<1) changes, the profits of retailer *ER*_2_ will not grow as the market share of blockchain-sensitive consumer *θ* increases.

### 6.3 Numerical analysis of comparison

This section mainly examines the comparison results between the optimal benefit of the *B* model and the *E* model due to blockchain technology and the situation without blockchain. We provide various numerical examples to demonstrate the reliability of our model.

#### 6.3.1 Numerical analysis between $t_{b}^{*}$ and $t_{e}^{*}$

**Fig 20:**
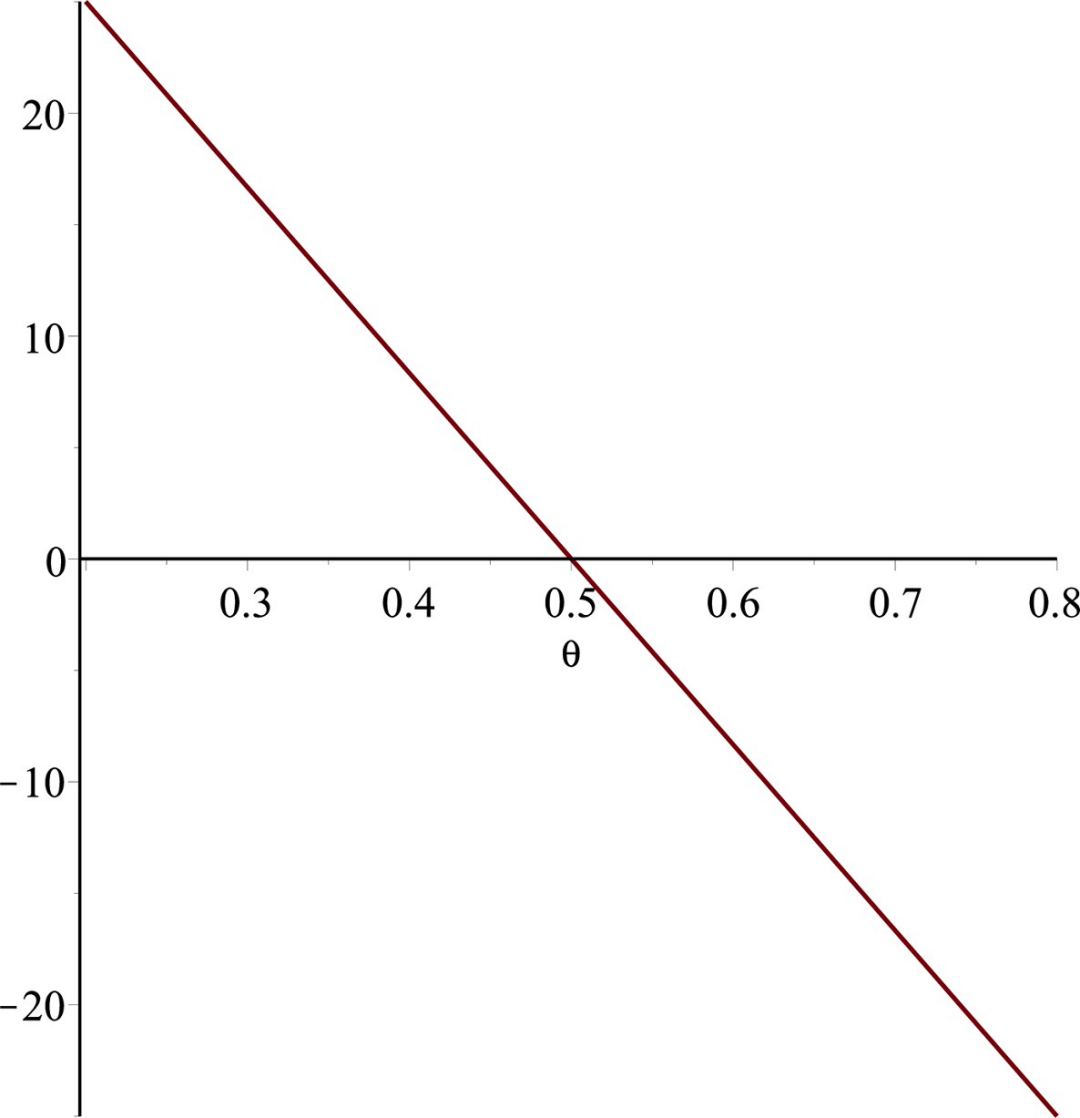
Relationship between $t_{b}^{*}-t_{e}^{*}$ and *θ*. (*a* = 100, *β* = 0.4).

To clearly present the impact of the market share of blockchain-sensitive consumer (*θ*) on $t_{b}^{*}-t_{e}^{*}$, the relationship between $t_{b}^{*}-t_{e}^{*}$ and *θ* will be presented by fixing *a* = 100, *β* = 0.4. As shown in Fig 20.

In Fig 20, setting *a* = 100, *β* = 0.4, we find that when the market share of blockchain-sensitive consumer *θ* (0<*θ*<1) is comparatively low, effort with blockchain of *ER*_1_ is greater than effort without blockchain of *ER*_2_; in contrast, when the market share of blockchain-sensitive consumer *θ* (0<*θ*<1) is comparatively high, effort without blockchain of *ER*_2_ is greater than effort with blockchain of *ER*_1_.

#### 6.3.2 Numerical analysis between $\Pi_{ER_{1}}^{B*}$ and $\Pi_{ER_{2}}^{E*}$

**Fig 21:**
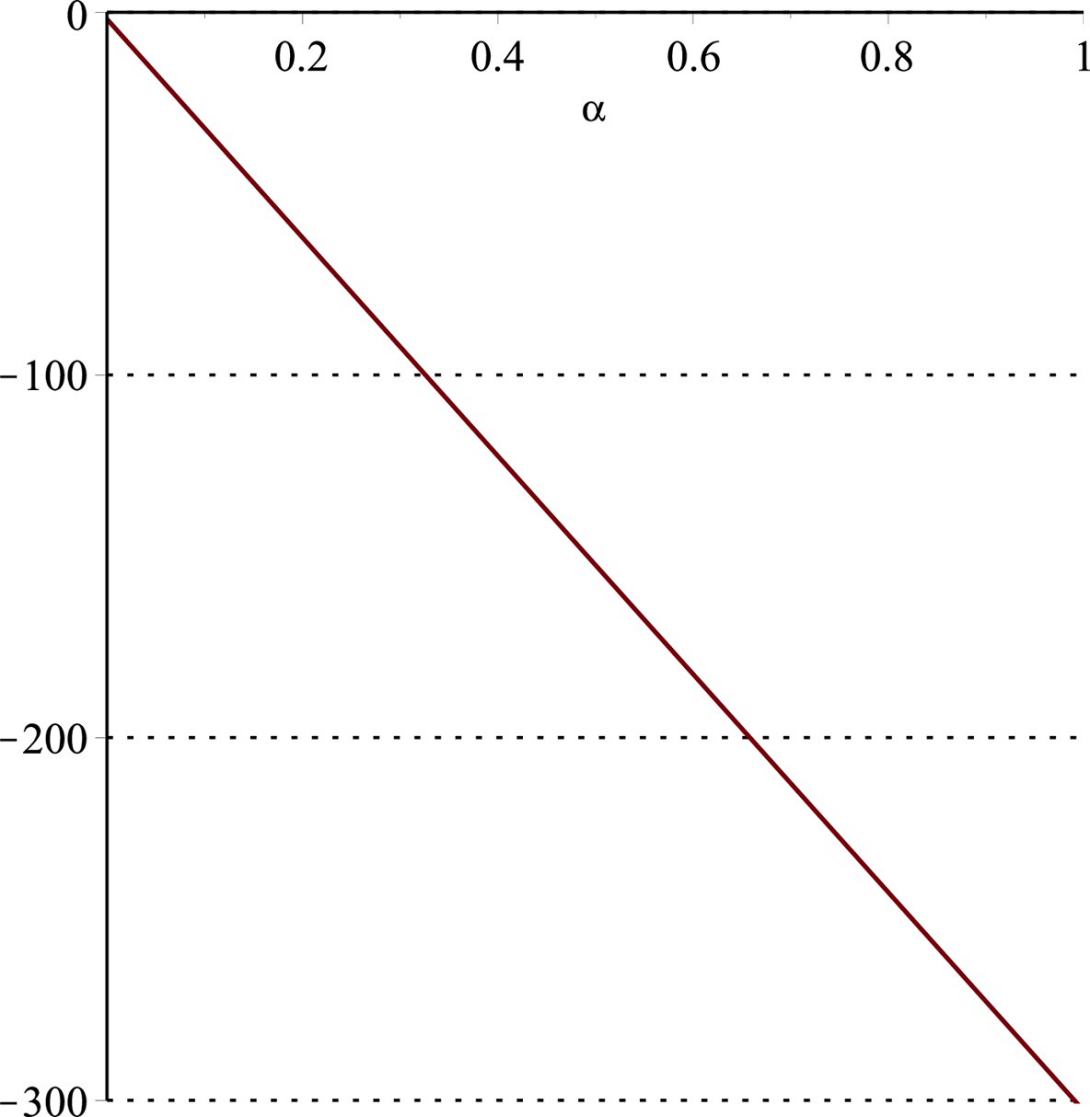
Relationship between $\Pi_{ER_{1}}^{B*}-\Pi_{ER_{2}}^{E*}$ and *α*.

**Fig 22:**
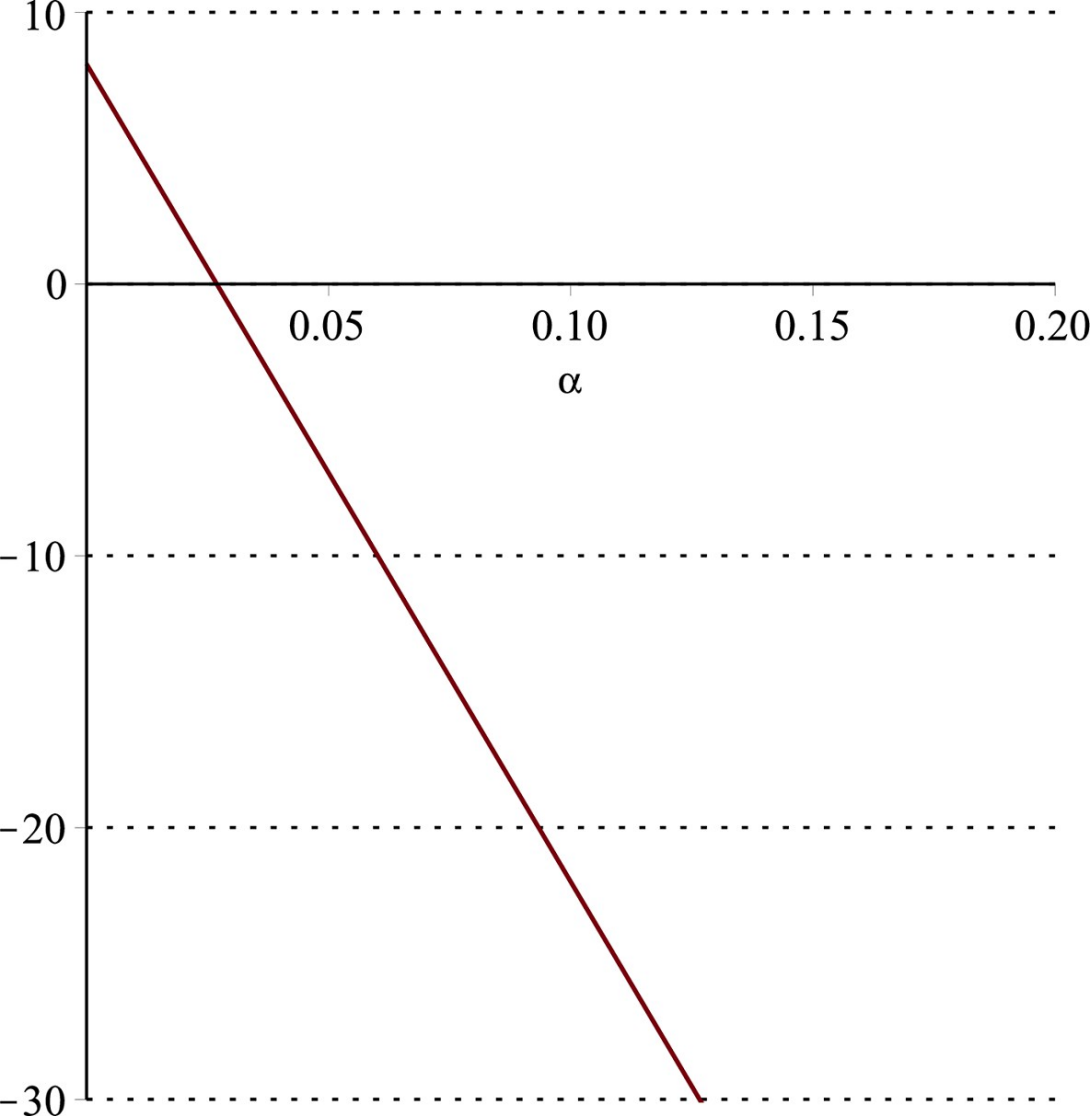
Relationship between $\Pi_{ER_{1}}^{B*}-\Pi_{ER_{2}}^{E*}$ and *α*. (*a* = 100, *p* = 5, *β* = 0.4, *θ* =0.4, *γ*_*b*_ = 10) (*a* = 100, *p* = 5, *β* = 0.4, *θ* =0.4, *γ*_*b*_ = 20).

**Fig 23:**
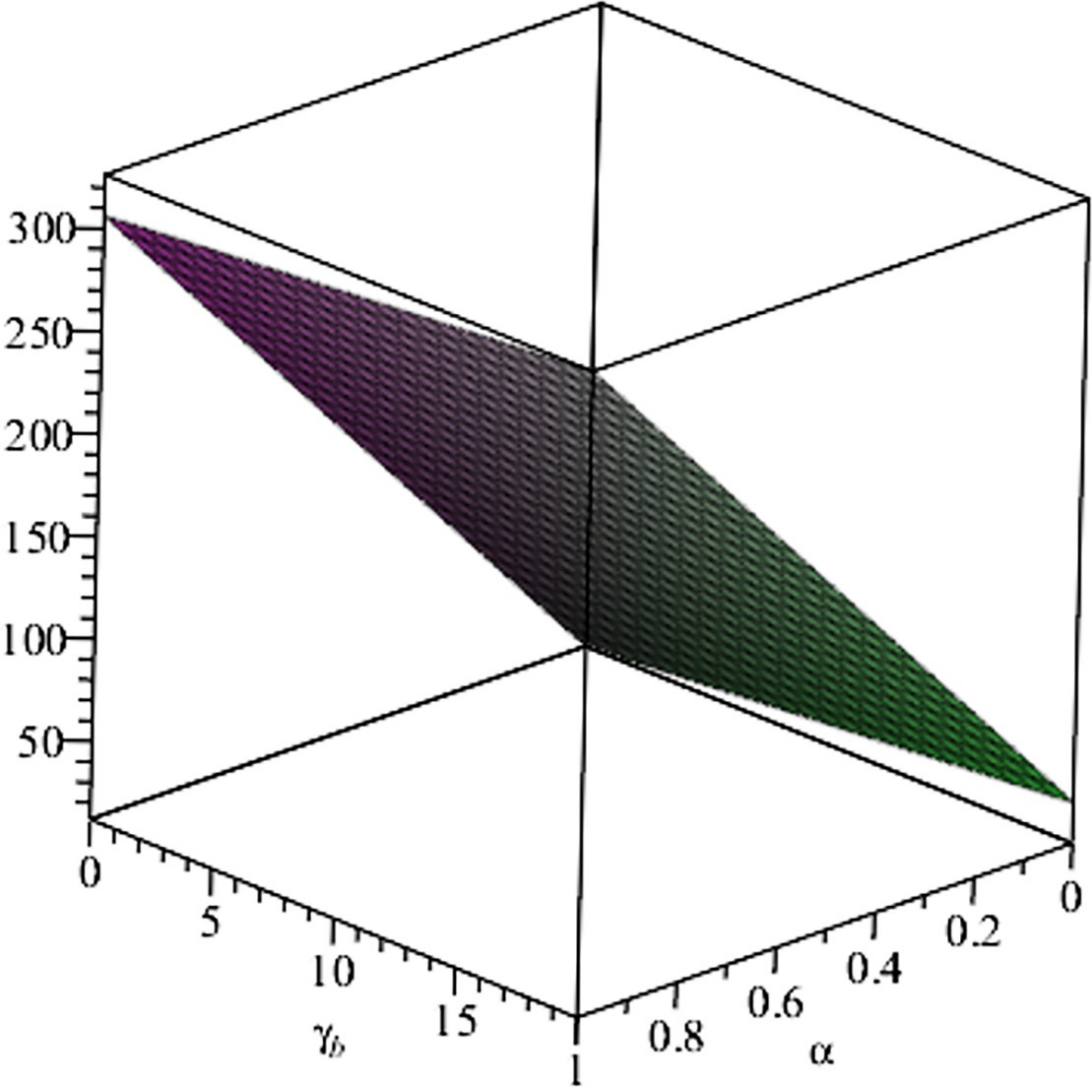
Relationship between $\Pi_{ER_{1}}^{B*}-\Pi_{ER_{2}}^{E*}$ and *α*/*γ*_*b*_. (*a* = 100, *p* = 5, *β* = 0.4, *θ* =0.6).

To clearly present the impact of the cost sensitivity to effort (*α*), cross-effort elasticity coefficient of demand (*β*) and the market share of blockchain-sensitive consumer (*θ*) on $\Pi_{ER_{1}}^{B*}-\Pi_{ER_{2}}^{E*}$, the relationship between $\Pi_{ER_{1}}^{B*}-\Pi_{ER_{2}}^{E*}$ and *α*, will be presented by fixing *a* = 100, *p* = 5, *β* = 0.4, *θ* =0.4/0.6, *γ*_*b*_ = 10/20. As shown in Figs 21–23.

Setting *a* = 100, *p* = 5, *β* = 0.4, *θ* =0.4, by comparing Figs 21 and 22, we find that when the market share of blockchain-sensitive consumer *θ* (*θ* = 0.4) and the brand value effect with blockchain *γ*_*b*_ (*γ*_*b*_ = 10) are comparatively low, no matter how *α* changes, the profits of *ER*_1_ are always less than the profits of *ER*_2_. However, when the market share of blockchain-sensitive consumer *θ* (*θ* = 0.4) is comparatively low, but the brand value effect with blockchain *γ*_*b*_ (*γ*_*b*_ = 20) is comparatively high, when *α* (0<*α*<0.0269) is low enough, the profits of *ER*_1_ are greater than the profits of *ER*_2_; when *α* (0.0269≤*α*<1) are not low enough, the profits of *ER*_1_ are less than the profits of *ER*_2_. In Fig 23, when the market share of blockchain-sensitive consumer *θ* (*θ* =0.6) is comparatively high, regardless of how the brand value effect with blockchain *γ*_*b*_ (*γ*_*b*_>0) or the cost sensitivity to effort *α* (0<*α*<1) changes, the profits of *ER*_1_ are always greater than the profits of *ER*_2_.

### 6.4 Numerical analysis of extended model

First, we suppose in this section that the values of the parameters are as follows: *a* = 100, *p* = 5, *p*_*s*_ = 4, *β* = 0.4, *θ* =0.4, *ξ*_1_ =0.3, and *ξ*_2_ =0.3. Then, we verify the effect of *α* (0<*α*<1) on $\Delta \frac{\partial \Pi_{S}}{\partial \alpha }$, $\Delta \frac{\partial \Pi_{ER_{1}}}{\partial \alpha }$, and $\Delta \frac{\partial \Pi_{ER_{2}}}{\partial \alpha }$ ($\Delta \frac{\partial \Pi_{S}}{\partial \alpha }=\frac{\partial \Pi_{S}^{A*}}{\partial \alpha }-\frac{\partial \Pi_{S}^{*}}{\partial \alpha }$, $\Delta \frac{\partial \Pi_{ER_{1}}}{\partial \alpha }=\frac{\partial \Pi_{ER_{1}}^{A*}}{\partial \alpha }-\frac{\partial \Pi_{ER_{1}}^{B*}}{\partial \alpha }$, and $\Delta \frac{\partial \Pi_{ER_{2}}}{\partial \alpha }=\frac{\partial \Pi_{ER_{2}}^{A*}}{\partial \alpha }-\frac{\partial \Pi_{ER_{2}}^{E*}}{\partial \alpha }$).

**Fig 24:**
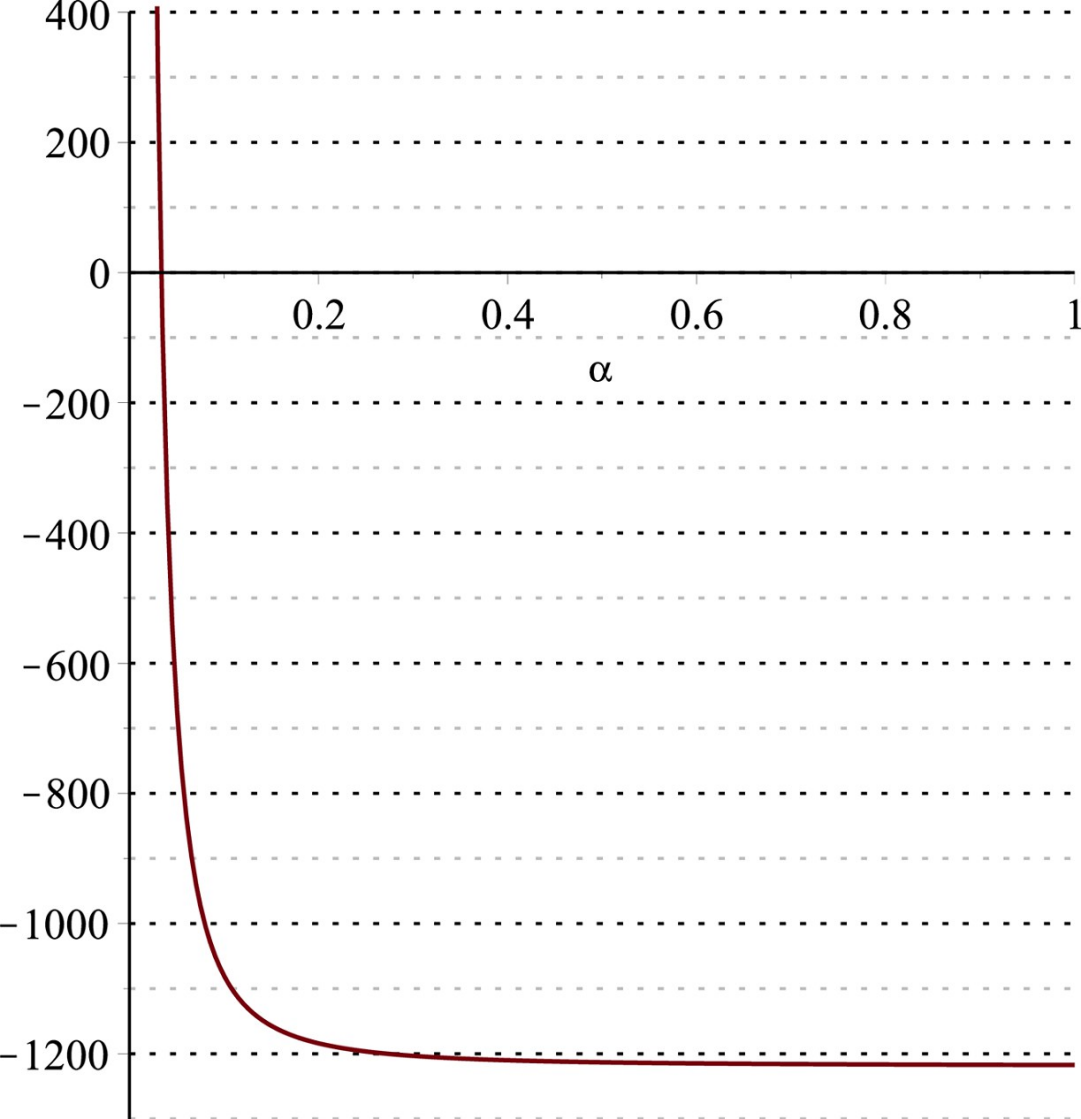
Relationship between $\Delta \frac{\partial \Pi_{S}}{\partial \alpha }$ and *α*.

**Fig 25:**
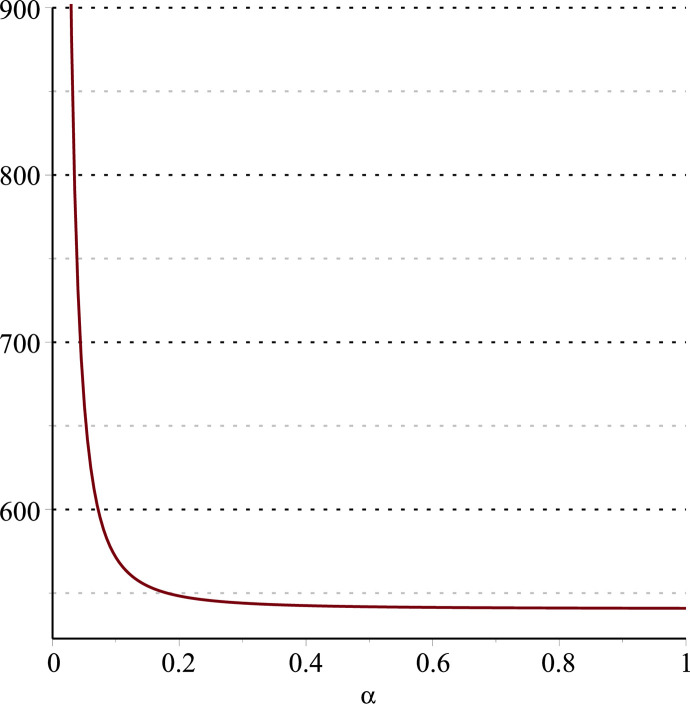
Relationship between $\Delta \frac{\partial \Pi_{ER_{1}}}{\partial \alpha }$ and *α*.

**Fig 26:**
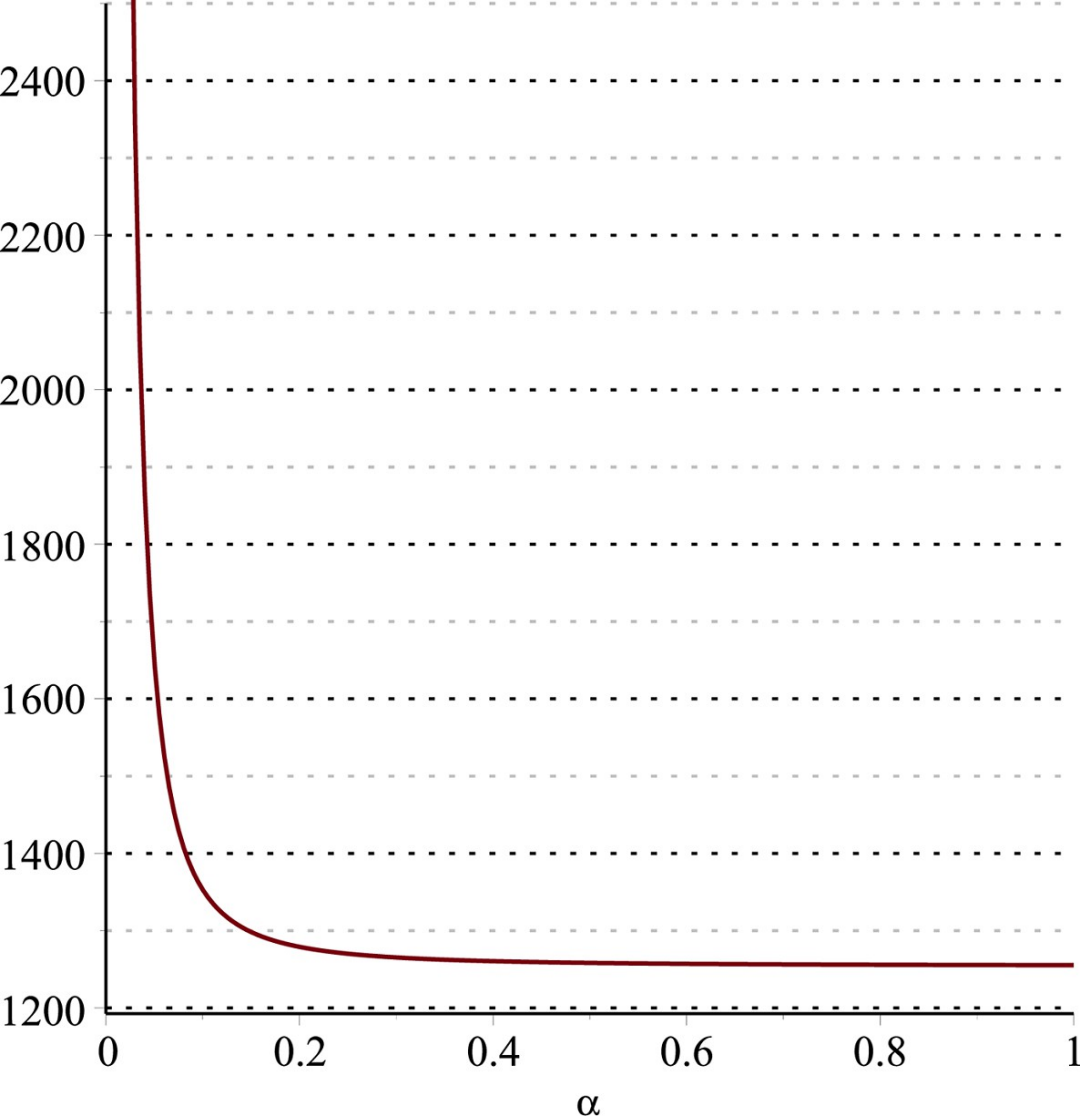
Relationship between $\Delta \frac{\partial \Pi_{ER_{2}}}{\partial \alpha }$ and *α*.

From Fig 24, we can easily find that when *α* (0<*α*<0.0338) is low enough, the value of $\Delta \frac{\partial \Pi_{S}}{\partial \alpha }$ is always positive; conversely, when *α* (0.0338≤*α*<1) is not low enough, the value of $\Delta \frac{\partial \Pi_{S}}{\partial \alpha }$ is negative. However, in Figs 25 and 26, we know that regardless of how *α* (0<*α*<1) changes, the values of $\Delta \frac{\partial \Pi_{ER_{1}}}{\partial \alpha }$ and $\Delta \frac{\partial \Pi_{ER_{2}}}{\partial \alpha }$ are always positive.

## 7. Discussion

### 7.1 Findings

In the context of the rapid development and application of blockchain, a new generation of information technology, this paper considers two competing e-tailers—a new type of e-tailer that implements blockchain technology and a traditional e-tailer that does not implement blockchain technology. We develop demand functions based on the heterogeneity of consumers’ sensitivity to blockchain technology under the two channels. Using the master-slave game model to obtain equilibrium solutions, we compare the optimal effort and the optimal profit of two e-tailers. For the basic properties, interestingly, we find that there exists a critical threshold on the cost sensitivity to effort that helps each e-tailer decide whether to implement effort. If the cost sensitivity to effort is high, two-sided e-tailers will reduce their effort as much as possible to obtain greater profits. Conversely, if the cost sensitivity to effort is low, they will increase their effort to obtain more benefits.

We also discuss the role of blockchain technology in competition between e-tailers and analyse the impact of the product brand effect brought by the traceability characteristic of blockchain on the competition between e-tailers. Specifically, (i) in the comparison of optimal effort, the results show that when the blockchain-sensitive consumer market accounted for less than half of the market share, the effort of new e-tailers who implemented blockchain technology would be greater than the effort of traditional e-tailers who did not implement blockchain technology; in contrast, it is less. (ii) In the comparison of optimal profit, the results show that when the market share of blockchain-sensitive consumers and the brand value effect with blockchain are both in a relatively small range or when the market share of blockchain-sensitive consumers is relatively small but the brand value effect with blockchain and the cost sensitivity to the effort are large, the new e-tailer’s profit is less than the traditional e-tailer’s profit. Under this circumstance, the implementation of blockchain technology is not a dominant choice for the new e-tailer. When the blockchain-sensitive consumer market has a relatively small market share but the brand value effect with blockchain is large, if the cost sensitivity to the effort is in a small range, the new e-tailer obtains more benefits. In other words, the implementation of blockchain technology is beneficial for the new e-tailer. However, when the blockchain-sensitive consumer market accounts for a relatively large market, no matter how the brand value effect with blockchain and the cost sensitivity to the effort are valued within their range, the profit level of the new e-tailer is always greater than that of the traditional e-tailer. That is, the implementation of blockchain technology is always a profitable choice for the new e-tailer.

In addition, in the robustness test of the extended model, we are happy to find that all core results are still valid, which gives us greater confidence to ensure the scientific accuracy of this paper. Finally, the most important finding is that e-tailers are always more profitable in the agency model regardless of the scope of the cost sensitivity to effort. In particular, when the cost sensitivity to effort is low enough, the agency model may be a more efficient way to improve the benefits of supply chain members. To present the findings of this paper more clearly, we summarise the findings in Table 7.

**Table 7:** Summary of the findings.

Findings	Logical proof	Explanation
The impact of blockchain technology adoption costs on companies	$↑(\sqrt{-\frac{B_{3}}{A_{3}}}\leq \alpha <1)$, $\Pi_{S}^{*}↑$;	**• If the cost sensitivity to effort is high**, managers should reduce their effort as much as possible to obtain greater profits.
	$↑(-\frac{B_{6}}{A_{6}}\leq \alpha <1)$, $\Pi_{ER_{1}}^{B*}↑$; $↑(-\frac{B_{11}}{A_{11}}\leq \alpha <1)$, $\Pi_{ER_{2}}^{E*}↑$.	**• If the cost sensitivity to effort is low**, they should increase their effort to obtain more benefits.
Conditions of use of blockchain technology	$\frac{1}{2}\leq \theta <1$, or if $0<\theta <\frac{1}{2}$ and $\gamma_{b}\geq \frac{2ap(1-\beta )(1-2\theta )}{\left(5-2\beta )(\beta +2\right)}$, $0<\alpha <-\frac{B_{16}}{A_{16}}$.	**• The blockchain-sensitive consumer market accounts for a relatively large proportion**, managers should adopt blockchain. **• The blockchain-sensitive consumer market accounts for a relatively small proportion,** the brand value effect with blockchain is relatively large and the cost sensitivity to the effort is small, managers should adopt blockchain.
What would be a better model to adopt	$\frac{\partial \Pi_{ER_{1}}^{A*}}{\partial \alpha }>\frac{\partial \Pi_{ER_{1}}^{B*}}{\partial \alpha }$; $\frac{\partial \Pi_{ER_{2}}^{A*}}{\partial \alpha }>\frac{\partial \Pi_{ER_{2}}^{E*}}{\partial \alpha }$.	**• When the cost sensitivity to effort is low enough,** the agency model may be a more efficient way to improve the benefits of supply chain members under certain conditions.

### 7.2 Theoretical contributions

This paper analyses the relevant properties and finds that the cost sensitivity to effort profoundly affects e-tailers’ effort implementation. Therefore, e-tailers should consider the extent of the cost sensitivity to effort when making effort implementation decisions. In particular, the if the cost sensitivity to effort is low, e-tailers should maximize their efforts to make more profits. Moreover, we find that whether e-tailers use blockchain technology depends on the brand spillover effect as well as the cost effect. In particular, e-tailers should implement blockchain technology when the brand spillover effect from blockchain technology is greater than the blockchain cost effect. In addition, when the blockchain-sensitive consumer market accounts for a relatively large market, no matter how the brand value effect with blockchain and the cost sensitivity to the effort are valued within their range, the profit level of the new e-tailer is always greater than that of the traditional e-tailer. Finally, e-tailers are always more profitable in the agency model regardless of the scope of the cost sensitivity to effort. That is, the agency model may be a more efficient way to improve the benefits of supply chain members.

### 7.3 Managerial insights

This section provides relevant managerial insights and hopes to help managers adopt blockchain technology more scientifically and optimize supply chain channels. Managerial insights can be summarized as follows:

First, managers should be aware that the use of blockchain technology cannot unconditionally benefit the platform. The use of blockchain is not free, the cost involves technical maintenance and product information collection, put information on the chain and update information, etc. Therefore, these costs due to the blockchain cannot be ignored by managers. Specifically, if the cost sensitivity to effort is high, managers should reduce their effort as much as possible to obtain greater profits. In contrast, if the cost sensitivity to effort is low, they should increase their effort to obtain more benefits.

Second, after incorporating the cost sensitivity to effort into consideration of the company’s operating cost, it was analysed together with relevant influencing factors. We find that the cost of using blockchain is closely related to the brand value effect it brings and the market share of blockchain-sensitive consumers. Furthermore, managers can use blockchain technology in the following conditions: the blockchain-sensitive consumer market accounts for a relatively large proportion, or although the blockchain-sensitive consumer market accounts for a relatively small proportion, the brand value effect with blockchain is relatively large and the cost sensitivity to the effort is small. Only under these conditions can blockchain technology help improve the company’s profit level.

Third, from another perspective, not using blockchain technology does not mean that companies will fall behind. Whether the use of blockchain can improve the company’s profit level depends on the combined effect of many factors. Companies that do not use blockchain technology can improve channel sales and increase profitability through marketing efforts. For example, improving customer experience before purchasing products, strengthening after-sales service, etc. Therefore, managers should evaluate specific business conditions and make scientific decisions under the premise of fully considering the combined effects of multiple factors on whether to use blockchain technology.

Finally, in the selection of sales scenarios, managers should choose more efficient scenarios to maximize profitability. Given this problem, the agency model may be a more efficient way to improve the benefits of supply chain members under certain conditions.

## 8. Conclusion

The research in this article shows that the use of blockchain technology is indeed a means to help companies gain a dominant position in a fiercely competitive environment, but whether e-platform retailers should blindly follow the trend and adopt this technology is worthy of in-depth discussion in a dual-channel supply chain. Our results show that in a dual-channel supply chain, blockchain technology can only provide value under certain specific conditions. This discovery provides a good reference for the development of dual-channel supply chains and the scientific application of blockchain technology, helping channel participants make better decisions for their retail channel.

However, this paper still has some limitations. Thus, there are several interesting directions for the future that deserve further expansion based on this paper. First, this article is based on the horizontal competition of e-tailers in a dual-channel supply chain in the context of blockchain. In fact, there are also some situations in which upstream suppliers implement blockchain technology. Therefore, incorporating upstream suppliers into the dual-channel supply chain to consider the issue of vertical competition with downstream retailers will be a further research direction. Second, while we find that the agency scenario in the extended model may be a more efficient choice, we do not optimize supply chain member profits from a vertical coordination perspective. Accordingly, developing a win–win coordination mechanism to achieve a profit-Pareto-improving situation among platforms, supplies and retailers may be our next point. Third, this paper regards retail price as an exogenous variable without considering its impact on basic demand. Therefore, we will introduce the retail price into the model as an endogenous variable to further study the impact of retail prices on the profits of all supply chain members in future work. Finally, we focus our analysis on the traceability of blockchain, while other ingredients, such as smart contracts and accurate delivery, can be further considered to investigate the blockchain’s advantages. Future research can examine their relationship together.

## Supporting information

S1 Appendix (DOCX)
