# Supplementary material for: Blockchain technology for supply chain traceability: A game-theoretic analysis between e-platforms
Source: PLoS One. 2024 Apr 25;19(4):e0297978. doi: 10.1371/journal.pone.0297978 (PMC11045145; doi:10.1371/journal.pone.0297978)
Supplement: S1 Appendix — (DOCX) [file pone.0297978.s001.docx]

**Appendix**

**1. Proof of Property 1**

(i) $\frac{\partial w^{*}}{\partial\alpha}=\frac{a(4+\beta\theta(2-\beta))}{2(5-2\beta)(1-\beta)(\beta+2)}>0$; (ii) $\frac{\partial w^{*}}{\partial\beta}=\frac{a((2\beta^{4}-8\beta^{3}+15\beta^{2}-20\beta+20)\theta+36+24\beta(1-\beta))\alpha+2p(\beta+2)^{2}(1-\beta)^{2}}{2(2\beta-5)^{2}(1-\beta)^{2}(\beta+2)^{2}}>0$; (iii) $\frac{\partial w^{*}}{\partial\theta}=\frac{a\alpha\beta(2-\beta)}{2(5-2\beta)(1-\beta)(\beta+2)}>0$.

**2. Proof of Property 2**

(i) $\frac{\partial t_{b}^{*}}{\partial\alpha}=-\frac{p}{(5-2\beta)\alpha^{2}}<0$; (ii) If $0<\alpha<-\frac{B_{1}}{A_{1}}$, therefore, $\frac{\partial t_{b}^{*}}{\partial\beta}=\frac{A_{1}\alpha+B_{1}}{2\alpha(5-2\beta)^{2}(1-\beta)^{2}(\beta+2)^{2}}>0$; If $-\frac{B_{1}}{A_{1}}\leq\alpha<1$, therefore,$\frac{\partial t_{b}^{*}}{\partial\beta}=\frac{A_{1}\alpha+B_{1}}{2\alpha(5-2\beta)^{2}(1-\beta)^{2}(\beta+2)^{2}}\leq0$; (iii) $\frac{\partial t_{b}^{*}}{\partial\theta}=-\frac{a(5-4\beta)(2-\beta)}{2(5-2\beta)(1-\beta)(\beta+2)}<0$. Where $A_{1}=a((8\beta^{4}-52\beta^{3}+135\beta^{2}-140\beta+40)\theta-8\beta^{4}+24\beta^{3}-42\beta^{2}+68\beta-78)$, $B_{1}=4p(\beta+2)^{2}(1-\beta)^{2}$.

**3. Proof of Property 3**

(i) $\frac{\partial t_{e}^{*}}{\partial\alpha}=-\frac{p}{(5-2\beta)\alpha^{2}}<0$; (ii) If $0<\alpha<-\frac{B_{2}}{A_{2}}$, therefore, $\frac{\partial t_{e}^{*}}{\partial\beta}=\frac{A_{2}\alpha+B_{2}}{2\alpha(5-2\beta)^{2}(1-\beta)^{2}(\beta+2)^{2}}>0$; If $-\frac{B_{2}}{A_{2}}\leq\alpha<1$, therefore, $\frac{\partial t_{e}^{*}}{\partial\beta}=\frac{A_{2}\alpha+B_{2}}{2\alpha(5-2\beta)^{2}(1-\beta)^{2}(\beta+2)^{2}}\leq0$; (iii) If $0<\beta<0.8672$, therefore, $\frac{\partial t_{e}^{*}}{\partial\theta}=\frac{a(4\beta^{2}-15\beta+10)}{2(5-2\beta)(1-\beta)(\beta+2)}>0$; If $0.8672\leq\beta<1$, therefore, $\frac{\partial t_{e}^{*}}{\partial\theta}=\frac{a(4\beta^{2}-15\beta+10)}{2(5-2\beta)(1-\beta)(\beta+2)}\leq0$. Where $A_{2}=a((-8\beta^{4}+60\beta^{3}-141\beta^{2}+140\beta-60)\theta-32\beta^{3}+96\beta^{2}-72\beta-28)$, $B_{2}=4p(\beta+2)^{2}(1-\beta)^{2}$.

**4. Proof of Property 4**

(i) If $0<\alpha<\sqrt{-\frac{B_{3}}{A_{3}}}$, therefore, $\frac{\partial\Pi_{S}^{*}}{\partial\alpha}=\frac{A_{3}\alpha^{2}+B_{3}}{4\alpha^{2}(5-2\beta)(1-\beta)(\beta+2)^{2}}<0$; If $\sqrt{-\frac{B_{3}}{A_{3}}}\leq\alpha<1$, therefore, $\frac{\partial\Pi_{S}^{*}}{\partial\alpha}=\frac{A_{3}\alpha^{2}+B_{3}}{4\alpha^{2}(5-2\beta)(1-\beta)(\beta+2)^{2}}\geq0$; (ii) If $0<\alpha<\frac{-B_{4}+\sqrt{(B_{4})^{2}-4A_{4}C_{4}}}{2A_{4}}$, therefore, $\frac{\partial\Pi_{S}^{*}}{\partial\beta}=\frac{A_{4}\alpha^{2}+B_{4}\alpha+C_{4}}{4\alpha(5-2\beta)^{2}(1-\beta)^{2}(\beta+2)^{3}}<0$; If $\frac{-B_{4}+\sqrt{(B_{4})^{2}-4A_{4}C_{4}}}{2A_{4}}\leq\alpha<1$, therefore, $\frac{\partial\Pi_{S}^{*}}{\partial\beta}=\frac{A_{4}\alpha^{2}+B_{4}\alpha+C_{4}}{4\alpha(5-2\beta)^{2}(1-\beta)^{2}(\beta+2)^{3}}\geq0$; (iii) If $0<\alpha<-\frac{B_{5}}{A_{5}}$, therefore, $\frac{\partial\Pi_{S}^{*}}{\partial\theta}=\frac{a(A_{5}\alpha+B_{5})}{2(5-2\beta)(1-\beta)(\beta+2)^{2}}<0$; If $-\frac{B_{5}}{A_{5}}\leq\alpha<1$, therefore, $\frac{\partial\Pi_{S}^{*}}{\partial\theta}=\frac{a(A_{5}\alpha+B_{5})}{2(5-2\beta)(1-\beta)(\beta+2)^{2}}\geq0$. Where $A_{3}=a^{2}((10-9\beta)(2-\beta)\theta^{2}+8\beta(3-2\beta)\theta+8\beta^{2}+4\beta+4)$, $B_{3}=-4p^{2}(\beta+2)^{2}(1-\beta)^{2}$;$\begin{matrix} A_{4}=a^{2}((-36\beta^{4}+231\beta^{3}-566\beta^{2}+580\beta-200)\theta^{2}+(64\beta^{4}-256\beta^{3}+464\beta^{2}-440\beta+240)\theta\\ -32\beta^{4}+32\beta^{3}-104\beta^{2}+192\beta+56) \end{matrix}$, $B_{4}=a(-8(\beta+2)(\beta^{2}+5)(1-\beta)^{2}\theta+16\beta^{5}+48\beta^{4}+20\beta^{3}-112\beta^{2}-108\beta+136)p$, $C_{4}=-12p^{2}(\beta+2)^{3}(1-\beta)^{2}$; $A_{5}=a(10-9\beta)(2-\beta)\theta+4\beta(3-2\beta)$, $B_{5}=-2\beta p(\beta+2)(1-\beta)$.

**5. Proof of Property 5**

(i) If $0<\alpha<-\frac{B_{6}}{A_{6}}$, therefore, $\frac{\partial\Pi_{ER_{1}}^{B*}}{\partial\alpha}=\frac{(A_{6}\alpha+B_{6})(A_{7}\alpha+B_{7})}{4\alpha^{2}(5-2\beta)^{2}(\beta+2)^{2}}<0$; If $-\frac{B_{6}}{A_{6}}\leq\alpha<1$, therefore, $\frac{\partial\Pi_{ER_{1}}^{B*}}{\partial\alpha}=\frac{(A_{6}\alpha+B_{6})(A_{7}\alpha+B_{7})}{4\alpha^{2}(5-2\beta)^{2}(\beta+2)^{2}}\geq0$; (ii) If $0<\alpha<-\frac{B_{8}}{A_{8}}$, therefore, $\frac{\partial\Pi_{ER_{1}}^{B*}}{\partial\beta}=\frac{(A_{8}\alpha+B_{8})(A_{9}\alpha+B_{9})}{\alpha(5-2\beta)^{3}(\beta+2)^{3}}<0$; If $-\frac{B_{8}}{A_{8}}\leq\alpha<1$, therefore, $\frac{\partial\Pi_{ER_{1}}^{B*}}{\partial\beta}=\frac{(A_{8}\alpha+B_{8})(A_{9}\alpha+B_{9})}{\alpha(5-2\beta)^{3}(\beta+2)^{3}}\geq0$; (iii) $\frac{\partial\Pi_{ER_{1}}^{B*}}{\partial\theta}=\frac{5a(2-\beta)(A_{10}\alpha+B_{10})}{2(5-2\beta)^{2}(\beta+2)^{2}}>0$. where $A_{6}=a((5\beta-10)\theta+2-4\beta)$, $B_{6}=2p(\beta+2)(1-\beta)$;$A_{7}=A_{6}=a((5\beta-10)\theta+2-4\beta)$, $B_{7}=-2p(\beta+2)(1-\beta)$; $A_{8}=A_{7}=A_{6}=a((5\beta-10)\theta+2-4\beta)$; $B_{8}=B_{7}=-2p(\beta+2)(1-\beta)$; $A_{9}=a((5\beta^{2}-20\beta+30)\theta-4\beta^{2}+4\beta-21)$, $B_{9}=3p(\beta+2)^{2}$; $A_{10}=a((10-5\beta)\theta-2+4\beta)$, $B_{10}=B_{6}$.

**6. Proof of Property 6**

(i) If $0<\alpha<-\frac{B_{11}}{A_{11}}$, therefore, $\frac{\partial\Pi_{ER_{2}}^{E*}}{\partial\alpha}=\frac{(A_{11}\alpha+B_{11})(A_{12}\alpha+B_{12})}{4\alpha^{2}(5-2\beta)^{2}(\beta+2)^{2}}<0$; If $-\frac{B_{11}}{A_{11}}\leq\alpha<1$, therefore, $\frac{\partial\Pi_{ER_{2}}^{E*}}{\partial\alpha}=\frac{(A_{11}\alpha+B_{11})(A_{12}\alpha+B_{12})}{4\alpha^{2}(5-2\beta)^{2}(\beta+2)^{2}}\geq0$; (ii) If $0<\alpha<-\frac{B_{13}}{A_{13}}$, therefore, $\frac{\partial\Pi_{ER_{2}}^{E*}}{\partial\beta}=\frac{(A_{13}\alpha+B_{13})(A_{14}\alpha+B_{14})}{\alpha(5-2\beta)^{3}(\beta+2)^{3}}<0$; If $-\frac{B_{13}}{A_{13}}\leq\alpha<1$, therefore, $\frac{\partial\Pi_{ER_{2}}^{E*}}{\partial\beta}=\frac{(A_{13}\alpha+B_{13})(A_{14}\alpha+B_{14})}{\alpha(5-2\beta)^{3}(\beta+2)^{3}}\geq0$; (iii) $\frac{\partial\Pi_{ER_{2}}^{E*}}{\partial\theta}=-\frac{a(10-3\beta)(A_{15}\alpha+B_{15})}{(5-2\beta)^{2}(\beta+2)^{2}}<0$. where $A_{11}=a((3\beta-10)\theta+8)$, $B_{11}=2p(\beta+2)(1-\beta)$;$A_{12}=A_{11}=a((3\beta-10)\theta+8)$, $B_{12}=-2p(\beta+2)(1-\beta)$; $A_{13}=A_{12}=A_{11}=a((3\beta-10)\theta+8)$; $B_{13}=B_{11}=2p(\beta+2)(1-\beta)$; $A_{14}=a((3\beta^{2}-20\beta+20)\theta+16\beta-4)$, $B_{14}=-3p(\beta+2)^{2}$; $A_{15}=A_{11}$, $B_{15}=B_{11}$.

**7. Proof of Property 9**

(i) $\frac{\partial t_{b}^{A*}}{\partial\alpha}=-\frac{p_{S}(\beta\xi_{2}+2\xi_{1})}{(\beta+2)(2-\beta)\alpha^{2}}<0$; (ii) $\frac{\partial t_{e}^{A*}}{\partial\alpha}=-\frac{p_{S}(\beta\xi_{1}+2\xi_{2})}{(\beta+2)(2-\beta)\alpha^{2}}<0$.

**8. Proof of Property 10**

(i) If $0<\alpha<\sqrt{-\frac{B_{19}}{A_{19}}}$, therefore, $\frac{\partial\Pi_{S}^{A*}}{\partial\alpha}=\frac{A_{19}\alpha^{2}+B_{19}}{\alpha(2-\beta)^{2}(\beta+2)^{2}}<0$; (ii) If $\sqrt{-\frac{B_{19}}{A_{19}}}\leq\alpha<1$, therefore, $\frac{\partial\Pi_{S}^{A*}}{\partial\alpha}=\frac{A_{19}\alpha^{2}+B_{19}}{\alpha(2-\beta)^{2}(\beta+2)^{2}}\geq0$. Where $A_{19}=A_{18}=A_{17}=a^{2}(\beta\theta-\beta-2\theta)^{2}$,

$\begin{matrix} B_{19}=-p_{S}^{2}((\xi_{1}^{2}+\xi_{2}^{2}-\xi_{1}-\xi_{2})\beta^{4}+(\xi_{1}\xi_{2}-\xi_{1}-\xi_{2})\beta^{3}+(-8\xi_{1}^{2}-7\xi_{2}^{2}+6\xi_{1}+6\xi_{2})\beta^{2} \\ +(-8\xi_{1}\xi_{2}+4\xi_{1}+4\xi_{2})\beta+12\xi_{1}^{2}+8\xi_{2}^{2}-8\xi_{1}-8\xi_{2}) \end{matrix}$.

**9. Proof of Property 11**

$\frac{\partial\Pi_{ER_{1}}^{A*}}{\partial\alpha}=\frac{(A_{20}\alpha+B_{20})(A_{21}\alpha+B_{21})}{\alpha^{2}(2-\beta)^{2}(\beta+2)^{2}}$. If $0<\xi_{1}<\frac{\beta}{2-\beta^{2}}$, $0<\xi_{2}<\frac{\xi_{1}(2-\beta^{2})}{\beta}$. When $0<\alpha<-\frac{B_{20}}{A_{20}}$, therefore, $A_{20}\alpha+B_{20}>0$; when $-\frac{B_{20}}{A_{20}}\leq\alpha<1$, therefore, $A_{20}\alpha+B_{20}\leq0$. If $0<\xi_{1}<\frac{\beta}{2-\beta^{2}}$, $0<\xi_{2}<\frac{\xi_{1}(2-\beta^{2})}{\beta}$. Therefore, $\forall\alpha\in\left(0,1\right)$，$A_{21}\alpha+B_{21}<0$. If $0<\xi_{1}<\frac{\beta}{2-\beta^{2}}$, $\frac{\xi_{1}(2-\beta^{2})}{\beta}\leq\xi_{2}<1$. Therefore, $\forall\alpha\in\left(0,1\right)$, $A_{20}\alpha+B_{20}<0$. If $0<\xi_{1}<\frac{\beta}{2-\beta^{2}}$, $\frac{\xi_{1}(2-\beta^{2})}{\beta}\leq\xi_{2}<1$. When $0<\alpha<-\frac{B_{21}}{A_{21}}$, therefore, $A_{21}\alpha+B_{21}>0$; when $-\frac{B_{21}}{A_{21}}\leq\alpha<1$, therefore, $A_{21}\alpha+B_{21}\leq0$.

**10. Proof of Property 12**

$\frac{\partial\Pi_{ER_{2}}^{A*}}{\partial\alpha}=\frac{(A_{22}\alpha+B_{22})(A_{23}\alpha+B_{23})}{\alpha^{2}(2-\beta)^{2}(\beta+2)^{2}}$. If $0<\xi_{2}<\frac{\beta}{2-\beta^{2}}$, $0<\xi_{1}<\frac{\xi_{2}(2-\beta^{2})}{\beta}$. When, $\forall\alpha\in\left(0,1\right)$, therefore, $A_{22}\alpha+B_{22}>0$. If $0<\xi_{2}<\frac{\beta}{2-\beta^{2}}$, $0<\xi_{1}<\frac{\xi_{2}(2-\beta^{2})}{\beta}$. When $0<\alpha<-\frac{B_{23}}{A_{23}}$, therefore, $A_{23}\alpha+B_{23}<0$; when $-\frac{B_{23}}{A_{23}}\leq\alpha<1$, therefore, $A_{23}\alpha+B_{23}\geq0$. If $0<\xi_{2}<\frac{\beta}{2-\beta^{2}}$, $\frac{\xi_{2}(2-\beta^{2})}{\beta}\leq\xi_{1}<1$. When $0<\alpha<-\frac{B_{22}}{A_{22}}$, therefore, $A_{22}\alpha+B_{22}<0$; when $-\frac{B_{22}}{A_{22}}\leq\alpha<1$, therefore, $A_{22}\alpha+B_{22}\geq0$. If $0<\xi_{2}<\frac{\beta}{2-\beta^{2}}$, $\frac{\xi_{2}(2-\beta^{2})}{\beta}\leq\xi_{1}<1$. When $\forall\alpha\in\left(0,1\right)$, therefore, $A_{23}\alpha+B_{23}>0$. Where $A_{22}=a(\beta\theta+2(1-\theta))$, $B_{22}=p_{S}((2-\beta^{2})\xi_{2}-\beta\xi_{1})$; $A_{23}=A_{22}=a(\beta\theta+2(1-\theta))$, $B_{23}=-p_{S}((2-\beta^{2})\xi_{2}-\beta\xi_{1})$.
